# Exploring the Effect of V_2_O_5_ and Nb_2_O_5_ Content on the Structural, Thermal, and Electrical Characteristics of Sodium Phosphate Glasses and Glass–Ceramics

**DOI:** 10.3390/ijms25053005

**Published:** 2024-03-05

**Authors:** Sara Marijan, Teodoro Klaser, Marija Mirosavljević, Petr Mošner, Ladislav Koudelka, Željko Skoko, Jana Pisk, Luka Pavić

**Affiliations:** 1 Division of Materials Chemistry, Ruđer Bošković Institute, Bijenička 54, 10000 Zagreb, Croatia; smarijan@irb.hr (S.M.); tklaser@irb.hr (T.K.); mmirosav@irb.hr (M.M.); 2 Department of General and Inorganic Chemistry, Faculty of Chemical Technology, University of Pardubice, 53210 Pardubice, Czech Republic; petr.mosner@upce.cz (P.M.); ladislav.koudelka@upce.cz (L.K.); 3 Department of Physics, Faculty of Science, University of Zagreb, Bijenička 32, 10000 Zagreb, Croatia; zskoko@phy.hr; 4 Department of Chemistry, Faculty of Science, University of Zagreb, Horvatovac 102a, 10000 Zagreb, Croatia; jana.pisk@chem.pmf.hr

**Keywords:** phosphate glasses, phosphate glass–ceramics, (micro)structure–property relationship, PXRD, SEM-EDS, EPR, vibrational spectroscopy, impedance spectroscopy, electrical properties

## Abstract

Na-V-P-Nb-based materials have gained substantial recognition as cathode materials in high-rate sodium-ion batteries due to their unique properties and compositions, comprising both alkali and transition metal ions, which allow them to exhibit a mixed ionic–polaronic conduction mechanism. In this study, the impact of introducing two transition metal oxides, V_2_O_5_ and Nb_2_O_5_, on the thermal, (micro)structural, and electrical properties of the 35Na_2_O-25V_2_O_5_-(40 − *x*)P_2_O_5_ − *x*Nb_2_O_5_ system is examined. The starting glass shows the highest values of DC conductivity, *σ*_DC_, reaching 1.45 × 10^−8^ Ω^−1^ cm^−1^ at 303 K, along with a glass transition temperature, *T*_g_, of 371 °C. The incorporation of Nb_2_O_5_ influences both *σ*_DC_ and *T*_g_, resulting in non-linear trends, with the lowest values observed for the glass with *x* = 20 mol%. Electron paramagnetic resonance measurements and vibrational spectroscopy results suggest that the observed non-monotonic trend in *σ*_DC_ arises from a diminishing contribution of polaronic conductivity due to the decrease in the relative number of V^4+^ ions and the introduction of Nb_2_O_5_, which disrupts the predominantly mixed vanadate–phosphate network within the starting glasses, consequently impeding polaronic transport. The mechanism of electrical transport is investigated using the model-free Summerfield scaling procedure, revealing the presence of mixed ionic–polaronic conductivity in glasses where *x* < 10 mol%, whereas for *x* ≥ 10 mol%, the ionic conductivity mechanism becomes prominent. To assess the impact of the V_2_O_5_ content on the electrical transport mechanism, a comparative analysis of two analogue series with varying V_2_O_5_ content (10 and 25 mol%) is conducted to evaluate the extent of its polaronic contribution.

## 1. Introduction

The rapid progression of battery technology is reshaping the landscape of energy storage, wherein sodium-ion batteries (SIBs) are emerging as a promising alternative to their lithium-ion counterparts. This shift is attributed to the cost-effectiveness, abundance, and widespread availability of sodium compared with lithium [1,2]. Despite the lower energy density resulting from sodium’s heavier mass, SIBs are being explored for grid-level applications where cost considerations take precedence over ultra-high energy density demands. In this context, a critical factor influencing the performance of SIBs is the cathode material [1,2]. The strategic design and development of such material, capable of delivering high capacity and voltage while maintaining electrochemical, thermal, and mechanical stability, significantly contribute to enhancing the energy density of the battery.

In the exploration of diverse cathode materials, particular emphasis is placed on systems incorporating both sodium and transition metal (TM) ions, such as V, Mn, Fe, Co, and Ni, among others. These combinations yield a range of materials, such as those with a 2D layered transition metal oxide (TMO) structure (e.g., NaCoO_2_, NaNi_0.5_Mn_0.5_O_2_) or polyanionic compounds featuring a 3D structure (e.g., NaFePO_4_, Na_3_V_2_(PO_4_)_3_) capable of hosting and releasing Na^+^ ions [1,2]. An additional advantage of Na–TM systems is their potential to exhibit mixed ionic–electronic conductivity, wherein the electronic contribution relies on the existence of a TM^n+^/TM^(n+1)+^ redox pair, adhering to the principles of the small polaron hopping (SPH) theory [3]. Each of the two contributions plays a significant role, with the redox centres facilitating charge transfer during the processes of Na^+^ intercalation/deintercalation and the ionic contribution promoting faster diffusion of Na^+^ ions [4].

Among the aforementioned cathode materials, NASICON-structured compounds stand out for their outstanding electrochemical performance, structural stability, and high ionic conductivity [4,5]. Particularly noteworthy are those derived from the Na-V-P-based system, such as Na_3_V_2_(PO_4_)_3_, which exhibits conductivity on the order of ~10^−8^ Ω^−1^ cm^−1^ at 298 K [6]. Nevertheless, its rate performance is significantly impeded by the intrinsic limitation of low electronic conductivity. Various strategies have been devised to tackle this challenge. Notably, doping with ions like Ni^2+^ [7], Nb^5+^ [8,9,10], Mo^6+^ [11], and W^6+^ [12] has emerged as an effective approach. This technique aids in the formation of V mixed valence states, thereby enhancing electronic conductivity. Furthermore, the partial substitution of Na and/or V with ions possessing larger radii can further promote Na^+^ mobility by expanding the migration channels for Na^+^. The addition of Nb^5+^ ions has proven particularly effective, markedly enhancing the intrinsic electronic conductivity and Na^+^ mobility in Na_3_V_2_(PO_4_)_3_, thereby reducing electrode polarization and enhancing rate capability [8,9,10].

In addition to Na-V-P crystalline materials, their glassy and glass–ceramic counterparts play a prominent role in the research and development of cathode materials. Noteworthy for their unique properties, these materials exhibit characteristics such as the absence of grain boundaries, isotropic conductivity, and exceptional compositional flexibility [13,14]. Moreover, Na-V-P glass and glass–ceramic systems provide an additional advantage with their capability to control the mechanism of electrical conductivity via straightforward adjustments in composition [15,16]. Specifically, research into the electrical properties of the ternary A-V-P system (A = Li, Na, K) revealed that the conductivity mechanism varies with the ratio *r* = [A_2_O]/([V_2_O_5_] + [P_2_O_5_]) [17,18,19,20,21]. Glasses featuring smaller *r* values demonstrate the highest conductivity and a predominantly polaronic mechanism, while those with higher *r* values tend to be predominantly ionic conductors and those in between display mixed ionic–electronic (polaronic) conductivity.

Building upon the aforementioned discoveries regarding both crystalline and glassy Na-V-P-based materials, and drawing on our proficiency in exploring the electrical properties of diverse alkali–TMO-containing phosphate-based systems manifesting distinct electrical conductivity mechanisms (ionic [22], polaronic [23], mixed ionic–polaronic [24,25,26]), we extended our research scope to encompass Na-V-P-based glass–(ceramic) systems incorporating Nb_2_O_5_. In our prior research, we examined the influence of Nb_2_O_5_ addition on the thermal, (micro)structural, and electrical characteristics of 35Na_2_O-10V_2_O_5_-(55 − *x*)P_2_O_5_ − *x*Nb_2_O_5_ (*x* = 0–40, mol%) glass and glass–ceramic series [27]. Solid-state impedance spectroscopy (SS-IS) measurements unveiled a purely ionic mechanism of electrical conductivity, despite the presence of 10 mol% of V_2_O_5_. Non-monotonic trends observed in DC conductivity and activation energy were ascribed to the facilitating effect of Nb_2_O_5_ on Na^+^ ion transport, indicating the mixed glass former effect (MGFE). Furthermore, it has been shown that this series mirrors the behaviour demonstrated by the majority of A-P-Nb (A = Li [28], Na [29,30,31,32,33], K [34]) glass and glass–ceramic systems, where the addition of Nb_2_O_5_ enhances both the thermal and electrical properties.

To further elucidate the impact of V_2_O_5_ content on the observed trends resulting from the gradual substitution of P_2_O_5_ with Nb_2_O_5_ in Na-V-P-based systems, we decided to investigate 35Na_2_O-25V_2_O_5_-(40 − *x*)P_2_O_5_ − *x*Nb_2_O_5_ (*x* = 0–35, mol%) glass and glass–ceramic series with higher V_2_O_5_ content. This study investigates the impact of introducing V_2_O_5_ and Nb_2_O_5_ on the glass-forming region along with the thermal, (micro)structural, and electrical properties of the given system. SS-IS reveals the initial glass as the optimal sample, showcasing the highest DC conductivity, *σ*_DC_ (1.45 × 10^−8^ Ω^−1^ cm^−1^ at 303 K), and glass transition temperature, *T*_g_ (371 °C). The incorporation of Nb_2_O_5_ gives rise to non-monotonic trends in both *σ*_DC_ and *T*_g_, with the lowest values at *x* = 20 mol%. Insights from vibrational spectroscopy indicate that these trends originate from the disruption of the mixed vanadate–phosphate network upon the addition of Nb_2_O_5_, thereby impeding polaronic transport. Using the model-free Summerfield scaling procedure, mixed ionic–polaronic conductivity is observed for *x* < 10 mol%, while for *x* ≥ 10 mol%, ionic conductivity prevails. A comparative analysis of two analogue Na-Nb-P series, with 10 mol% V_2_O_5_ [27] and 25 mol% V_2_O_5_, evaluates the impact of V_2_O_5_ content on the electrical transport mechanism and the extent of its polaronic contribution.

## 2. Results and Discussion

The glass-forming region (GFR) in the quaternary system 35Na_2_O-25V_2_O_5_-(40 − *x*)P_2_O_5_ − *x*Nb_2_O_5_ (25V series) is observed to be relatively wide, incorporating Nb_2_O_5_ up to 25 mol%. Beyond the GFR, spontaneous crystallization occurs, leading to the formation of partially crystallized (glass–ceramic) samples, 25V-30Nb and 25V-35Nb. Table 1 presents the batch compositions of the prepared glasses and glass–ceramics, accompanied by selected physical properties.

### 2.1. General Properties and Thermal Behaviour

**Figure 1:**
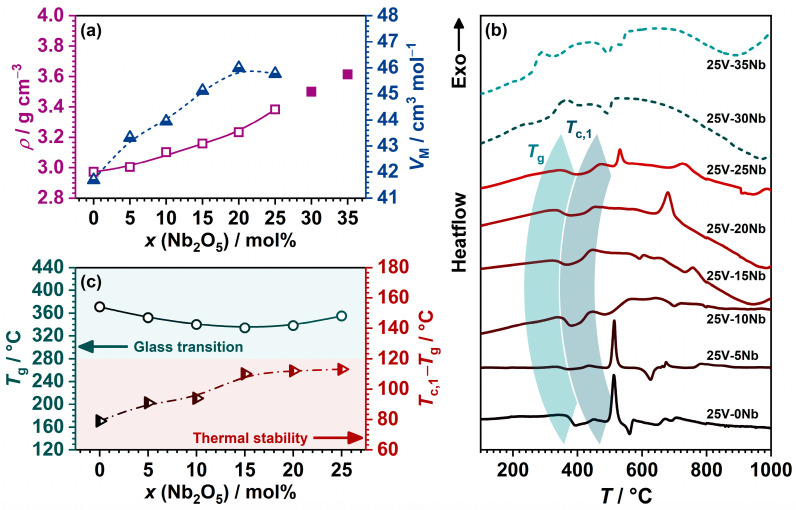
(**a**) Dependence of density, *ρ*, and molar volume, *V*_M_, on Nb_2_O_5_ content; (**b**) DTA curves; and (**c**) dependence of glass transition temperature, *T*_g_, and the parameter (*T*_c_ − *T*_g_) on Nb_2_O_5_ content. The lines connecting data points in (**a**,**c**) are a guide for the eye.

The density, *ρ*, of the samples demonstrates an increasing trend with the addition of Nb_2_O_5_; see Figure 1a. Interestingly, the molar volume, *V*_M_, exhibits a non-monotonic pattern as the content of Nb_2_O_5_ increases, reaching its peak for glass at 25V-20Nb; see Figure 1a. This observed behaviour is surprising, given that *V*_M_ typically follows the opposite trend to that of *ρ* [29,30,31,32,33,34]. Changes in *ρ* and *V*_M_ are commonly attributed to two factors: (i) the difference in molar masses—Nb_2_O_5_ (265.81 g/mol) having a greater molar mass than P_2_O_5_ (141.943 g/mol) and (ii) the difference in bond strengths—Nb–O bond being stronger (772 kJ/mol [35]) compared to the P–O bond (599 kJ/mol [35]). While these factors straightforwardly account for the increasing trend in *ρ*, comprehending the unconventional *V*_M_ trend is more intricate. This phenomenon can be explained as a result of incorporating Nb_2_O_5_ in the form of voluminous octahedral NbO_6_ structural units [36], which disrupt the initially compact mixed vanadate–phosphate (V-P) network, where vanadate and phosphate units are mutually interconnected via V–O–P bonds. This transformation leads to a more “open” network, as indicated by the rising *V*_M_ values.

The DTA curves for each sample exhibit an endothermic effect indicative of the glass transition, *T*_g_, along with one or more exothermic signals associated with the crystallization process, *T*_c_; see Figure 1b. The temperature of the onset of *T*_g_ and the peak temperature of the first *T*_c_ for all the samples are determined and listed in Table 1, along with the quantity (*T*_c_ − *T*_g_), representing the thermal stability (TS) of the glasses. The dependence of *T*_g_ on Nb_2_O_5_ content in the 25V series is illustrated in Figure 1c. Similar to *V*_M_, the *T*_g_ trend follows a non-monotonic pattern, gradually decreasing until reaching a minimum for the 25V-20Nb glass, after which it begins to rise. These findings diverge from the anticipated thermal behaviour observed in similar glass systems, where *T*_g_ typically increases nearly linearly with the addition of Nb_2_O_5_ [27,29,30,31,32,33,34]. In contrast to the anticipated rise in *T*_g_ with the incorporation of Nb_2_O_5_, the 25V series displays a minimum, and *T*_g_ values exhibit only small changes upon the introduction of Nb_2_O_5_.

Variations in *T*_g_ typically arise from the same factors influencing *ρ* and *V*_M_, such as the replacement of weaker P–O bonds with stronger Nb–O bonds and the formation of a more condensed mixed niobate–phosphate (Nb-P) network, leading to an increase in *T*_g_ in previously reported series [27,29,30,31,32,33,34]. However, the non-monotonic trend in *T*_g_ within the 25V series indicates the unfavourable influence of Nb_2_O_5_ on *T*_g_. As elucidated concerning *V*_M_, this behaviour stems from the incorporation of niobate units that disrupt the initial mixed V-P network. As the predominantly mixed niobate–vanadate (Nb-V) network forms for *x* > 20 mol%, a slight elevation in *T*_g_ is observed, with the addition of more Nb_2_O_5_ exerting a positive influence on the *T*_g_ values. On the contrary, the TS of the glasses, assessed through the quantity (*T*_c_ − *T*_g_) [37], increases upon the initial incorporation of Nb_2_O_5_ up to 15 mol% and stabilizes for glasses with 15 ≤ *x* ≤ 25 (see Figure 1c), revealing a beneficial influence of Nb_2_O_5_ on the TS of the investigated glass compositions. Moreover, the DTA curves of the partially crystalline samples, 25V-30Nb and 25V-35Nb, reveal a glass transition. The *T*_g_ values associated with these transitions are markedly lower in comparison with the glasses within the same series. As detailed later in the text, both glass–ceramics feature significant amounts of the Na_13_Nb_35_O_94_ crystalline phase. Thus, it can be inferred that the diminished *T*_g_ values stem from the residual glassy matrix undergoing depletion of sodium and niobium, which participate in the formation of a confirmed crystalline phase.

### 2.2. PXRD and SEM-EDS Analysis

**Figure 2:**
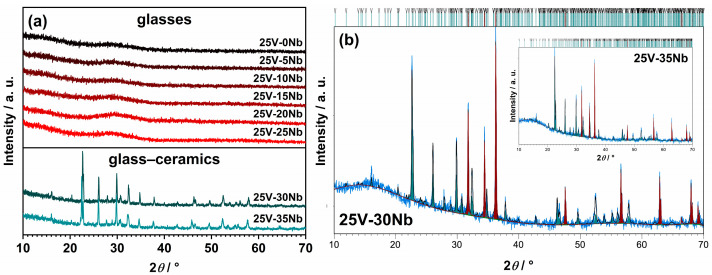
(**a**) PXRD patterns of glasses and glass–ceramics from this study; (**b**) Rietveld refinement of 25V-30Nb and 25V-35Nb glass–ceramics mixed with powder of ZnO used as an internal standard for amorphous phase quantification. Experimental data are given by the blue line, the calculated pattern is shown in black, teal vertical marks show the positions of diffraction lines belonging to the dominant Na_13_Nb_35_O_94_ phase, while the positions of ZnO lines are given as maroon vertical marks.

The PXRD method validates the successful preparation of glasses over a broad composition range (0 ≤ *x* ≤ 25), indicating their amorphous nature as evidenced by the absence of diffraction maxima, with only the characteristic amorphous “halo” present in the diffraction patterns; see Figure 2a. However, in melts with higher Nb_2_O_5_ amounts (*x* ≥ 30), spontaneous crystallization occurs during cooling, yielding glass–ceramic, a composite material consisting of both crystalline and amorphous phases. Both partially crystallized samples, 25V-30Nb and 25V-35Nb, contain a crystalline Na_13_Nb_35_O_94_ phase (24882-ICSD) [38] in addition to the amorphous phase. Quantitative phase analysis of both glass–ceramics performed using the Rietveld refinement method shows the 25V-30Nb sample contains 29 wt.% of the crystalline phase Na_13_Nb_35_O_94_, with the remaining 71 wt.% being amorphous glass matrix; see Figure 2b. With further introduction of Nb_2_O_5_, the proportion of the crystalline phase Na_13_Nb_35_O_94_ embedded into the glass matrix for the 25V-35Nb glass–ceramic sample increases to 45 wt.%.

**Figure 3:**
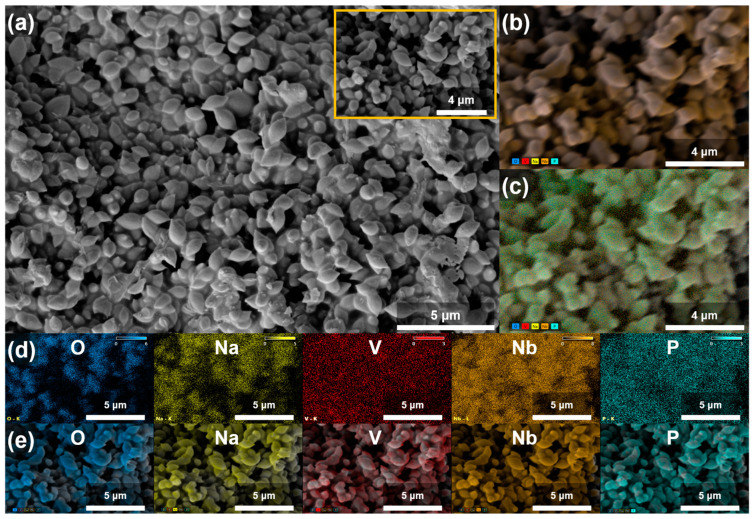
(**a**) SEM micrographs of 25V-30Nb glass–ceramic; (**b**) count X-ray mapping; (**c**) quantitative X-ray mapping; (**d**) count and (**e**) quantitative elemental mapping of O, Na, V, Nb, and P from a selected area of the given glass–ceramic.

SEM-EDS microscopy is employed to gain insight into the morphology and elemental composition of the prepared samples. In the case of the glassy samples, the SEM micrograph confirms the amorphous structure and uniform distribution of the elements. Conversely, for the partially crystallized ones obtained by spontaneous crystallization beyond the GFR boundary, the SEM micrograph reveals areas with distinct microstructural characteristics. As illustrated in Figure 3a, the 25V-30Nb glass–ceramic features grains that are uniformly embedded in the amorphous glassy matrix. These grains assume the shape of spheres, with a diameter ranging from ~500 nm to ~2 μm.

Furthermore, the SEM micrograph of the 25V-35Nb glass–ceramic shown in Figure S1 in the Supplementary Materials (SM) reveals that with the addition of more Nb_2_O_5_, the morphology further develops with the ongoing crystallization process. Fused grains take on the form of plate-shaped grains, varying in size from approximately 2 to 20 μm in diameter. These grains are also uniformly embedded in the remaining amorphous glassy matrix.

The EDS maps depicted in Figure 3b,c and Figure S1b,c in the SM visually illustrate the distribution and variation of the chemical elements across the two glass–ceramic samples. Examination of the maps for the individual elements O, Na, V, Nb, and P reveals distinct compositional differences between the grains and the residual glass matrix area, as shown in Figure 3d,e and Figure S1d,e in the SM. In the grain areas, the elements Na, Nb, and O are concentrated, while V and P are present only in trace amounts on the surface. This observation aligns with the EDS analysis (see Figure S1f in the SM), which indicates that the composition of the observed grains closely matches that of the Na_13_Nb_35_O_94_ crystalline phase. As the identified crystalline phase progressively crystallizes with the increasing Nb_2_O_5_ content, the amorphous glass matrix is depleted of Na, Nb, and O elements, while the elements V and P remain in the glass matrix. This is particularly evident in the 25V-35Nb sample (see Figure S1c in the SM), where the EDS analysis reveals that the composition of the grains (area 2) corresponds to that of the Na_13_Nb_35_O_94_ crystalline phase, while the glassy matrix (area 3) becomes enriched in V, as illustrated in Figure S1g in the SM.

### 2.3. Vibrational Spectroscopy—Raman and IR-ATR Studies

**Figure 4:**
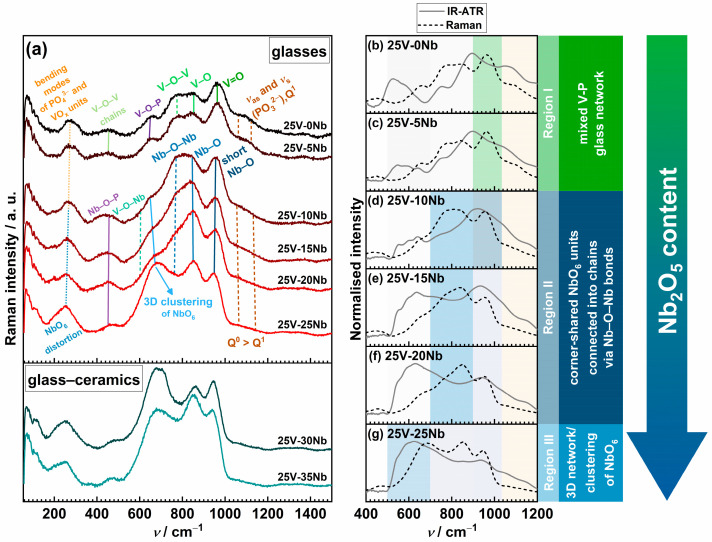
(**a**) Raman spectra of glasses and glass–ceramics from the 25V series and (**b**–**g**) comparison of IR-ATR and Raman spectra for individual glass samples. Shading in (**b**–**g**) corresponds to the band assignment in (**a**). The orange shade denotes the vibration of phosphate units, the green shade represents vanadate units, and the blue shade indicates niobate units. The most intense colored regions correspond to the dominant signals.

The effect of the gradual substitution of P_2_O_5_ with Nb_2_O_5_ on the structural characteristics of the glass and glass–ceramic samples from the studied 25V series is investigated using two complementary vibrational spectroscopy techniques—Raman and attenuated total reflectance infrared (IR-ATR) spectroscopy. Figure 4 illustrates the changes in Raman and IR-ATR spectra resulting from the incorporation of Nb_2_O_5_, and a detailed list of observed bands and their assignments, sourced from the literature data [29,30,31,32,33,34,36,39,40,41,42,43,44,45,46,47,48,49,50,51,52,53,54,55,56,57,58,59,60,61,62], can be found in Table S1 in the SM.

The Raman and IR-ATR spectra for the initial 25V-0Nb glass reveal a mixed glass network composed of phosphate and vanadate units. The bands observed between 1045 and 1285 cm^−1^ indicate the presence of Q^2^ and Q^1^ phosphate units, while the most prominent signal, occurring at ~965 cm^−1^, originates from the symmetric stretching vibrations of the double V=O bond. The latter band may also include a signal corresponding to isolated Q^0^ phosphate units, whose prevalence is expected for the O/P ratio of 4.5; see Table 1. However, the strong Raman scattering tendency of transition metal oxides typically masks the Raman response from phosphate units, thereby concealing the Q^0^ signals beneath those originating from the vanadate units. Furthermore, the signal at ~850 cm^−1^ signifies the stretching of the V–O bonds within VO_x_ units, whereas the signal at ~750 cm^−1^ corresponds to the stretching of the bridging V–O–V bonds within mutually interconnected VO_x_ units. The presence of a band at ~640 cm^−1^, associated with V–O–P bonding, confirms the formation of a mixed V-P glass network. Additionally, the signal at ~470 cm^−1^ aligns with the stretching vibrations of the V–O–V bonds in metavanadate chains, suggesting a substantial level of crosslinking among the vanadate structural units.

The changes in Raman spectra resulting from the incorporation of Nb_2_O_5_ are depicted in Figure 4a. Upon the initial introduction of Nb_2_O_5_ (*x* = 5), only a subtle increase in the intensity of the signal at 965 cm^−1^ is observed, and the spectra of the two glasses, 25V-0Nb and 25V-5Nb, exhibit nearly identical features. More significant changes in the spectra become evident for *x* ≥ 10, as the intensities of the bands within the 520–1030 cm^−1^ range progressively increase. The most noticeable alteration in shape occurs in the cases of 25V-15Nb and 25V-20Nb glasses, where the signal at ~850 cm^−1^ emerges as the strongest one. In contrast, the shape of the Raman spectrum of the 25V-10Nb glass closely resembles those of the starting 25V-0Nb glass and 25V-5Nb glasses, suggesting that this composition marks the point where the structural transition occurs. The spectrum of the 25V-25Nb glass differs from the others, as the signal at ~680 cm^−1^ exhibits a significant increase in intensity, establishing itself as the dominant signal alongside the one at ~850 cm^−1^. The changes in spectral shape and signal intensities observed with the increasing content of Nb_2_O_5_ can be ascribed to distinct structural motifs formed by Nb_2_O_5_ within the glass structure [32,33,34,61]. These structural features give rise to vibrations categorized into three distinct domains: (i) short Nb–O bonds within highly distorted NbO_6_ units (900–1030 cm^−1^, Domain I), (ii) NbO_6_ octahedra linked into chains via Nb–O–Nb bonds (750 to 900 cm^−1^, Domain II), and (iii) 3D network of less distorted corner-shared NbO_6_ units (520–750 cm^−1^, Domain III).

As mentioned earlier, in the case of glasses with 10 ≤ *x* ≤ 20, the most prominent signal is observed within Domain II. Despite the overlap with signals attributed to the V–O bonds in VO_x_ units, its intensity consistently amplifies with rising Nb_2_O_5_ content, thus suggesting that niobate units predominantly participate in the formation of a chain-like structure. Moreover, a band at approximately ~450 cm^−1^, indicative of P–O–Nb bonding, becomes more prominent, thereby confirming cross-linking between the PO_4_ and NbO_6_ units. However, with the addition of more Nb_2_O_5_, the intensity of this band gradually decreases. Meanwhile, the signal within Domain III broadens and strengthens, suggesting a rising number of less-distorted corner-shared NbO_6_ units connected into chains via Nb–O–Nb bonds. Additionally, it is worth noting that signals arising from mixed V–O–Nb bridging bonds may exist in this range; nevertheless, the overlapping signals describing distinct vibration modes of the vanadate and niobate units make their detection challenging. In the spectrum of the 25V-25Nb glass, a change in the intensity ratio between the two maxima in Domains II and III occurs, with the signal in Domain III becoming the strongest, indicative of the predominant participation of NbO_6_ units in the formation of 3D clusters. The Raman spectra of the 25V-30Nb and 25V-35Nb glass–ceramics further highlight a pronounced presence of the signal in Domain III. This observation is anticipated, as both compositions encompass substantial amounts of the Na_13_Nb_35_O_94_ crystal phase, distinguished by its crystal structure featuring a 3D network of edge- and corner-shared NbO_6_ octahedra.

According to these findings, the investigated glasses–(ceramics) can be categorized into three compositional regions based on their structural characteristics: (i) a predominantly mixed V-P network (*x* < 10, Region I); (ii) a mixed Nb-V-P glass network (10 ≤ *x* ≤ 20, Region II), where NbO_6_ units participate in the formation of chains through Nb–O–Nb bonds; and (iii) a predominantly mixed Nb-V network (*x* ≥ 25, Region III), where NbO_6_ units tend to cluster, forming a 3D network (see Figure 4b–g).

The results of IR-ATR analysis reveal a structural evolution similar to that observed in the Raman spectra (see Figure 4b–g), showcasing a transition from a predominantly mixed V-P network to a predominantly mixed Nb-V network upon the substitution of P_2_O_5_ with Nb_2_O_5_. Notably, a majority of the bands are present in both the IR-ATR and Raman spectra. Nevertheless, while the Raman spectra are characterized by strong, narrow bands linked to symmetric stretching vibrations, the IR-ATR spectra take on a more complex form, where broad yet intense bands, attributed to asymmetric stretching and bending motions, prevail [62,63]. This is apparent when comparing the Raman and IR-ATR spectra of the initial 25V-0Nb glass (see Figure 4b), where the two strongest signals in the IR-ATR spectrum correspond to the deformation modes of the phosphate and vanadate units and the asymmetric stretching of the bridging oxygen atoms. Because Raman spectra often obscure signals associated with phosphate units beneath those emanating from TMO polyhedra owing to their notably greater polarizability [33,34], utilising IR-ATR spectroscopy, which preserves information concerning phosphate units, serves as an additional avenue for achieving a more comprehensive understanding of the structure of the studied glasses.

### 2.4. Electrical Properties

#### 2.4.1. Complex Impedance Plane and Direct Current (DC) Conductivity

**Figure 5:**
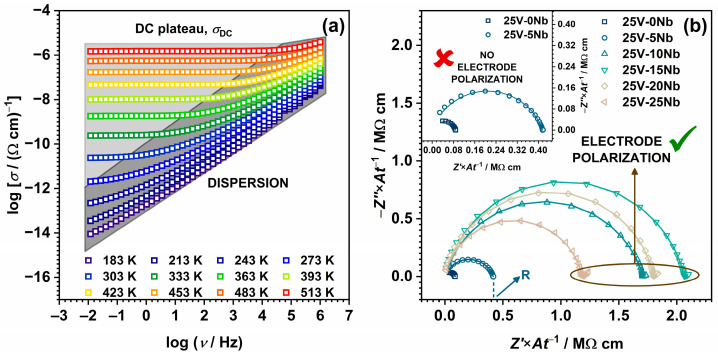
(**a**) Conductivity spectra at different temperatures for 25V-20Nb glass and (**b**) the complex impedance spectra for all glass samples at 483 K. Empty symbols represent the experimental data, while the lines depict the fit obtained through EEC modelling.

Figure 5a presents the conductivity spectra at various temperatures for glass 25V-20Nb, chosen as a representative for the entire series. The spectra reveal two distinct regions: (i) a frequency-independent segment referred to as the DC plateau, *σ*_DC_, which is linked to the long-range motion of charge carriers, and (ii) a frequency-dependent region (AC part) observed at lower temperatures and higher frequencies, i.e., the dispersion that arises from the localized motion of charge carriers over shorter distances. Additionally, upon a thorough examination of the conductivity spectra, it is evident that glasses where *x* ≥ 10 exhibit a slight decline in conductivity at the highest temperatures and the lowest frequencies, which points to the emergence of the electrode polarization effect. This phenomenon, indicative of ionic conductivity, arises due to the accumulation of mobile Na^+^ ions at the surface of the blocking gold electrodes and is further validated through the analysis of the complex impedance spectra depicted in Figure 5b.

Specifically, the presentation of experimental data through complex impedance spectra provides a comprehensive insight into the diverse processes in the electrical response of the investigated samples, with all impedance spectra analysed by modelling with the corresponding electrical equivalent circuit (EEC) using the complex non-linear least-squares (CNLLSQ) method. At 303 K, the impedance spectra for all samples exhibit a depressed semicircle, indicating the bulk process where the intercept on the *Z*’ axis corresponds to the DC resistance, *R*, at the given temperature. These impedance spectra are described using the EEC model featuring a parallel combination of the resistor (R) and a constant phase element (CPE). However, at higher temperatures, the formation of an additional low-frequency “spur” can be observed in the impedance spectra of samples where *x* ≥ 10, a characteristic typical of ionically conducting glasses, which corresponds to the aforementioned electrode polarization effect. The EEC model for these spectra comprises a parallel R–CPE combination connected in series with the CPE. As illustrated in Figure 5b, the experimental data closely align with the theoretical curves, and the DC conductivity, *σ*_DC_, is calculated using the formula *σ*_DC_ = *t*/(*A* × *R*), where *t* is the sample thickness, *A* is the electrode area, and *R* is the electrical resistance. The corresponding *σ*_DC_ values at 303 K, listed in Table 2, demonstrate close agreement between the DC conductivity derived from the DC plateau and the values obtained through EEC modelling.

#### 2.4.2. DC Conductivity and Activation Energy

**Figure 6:**
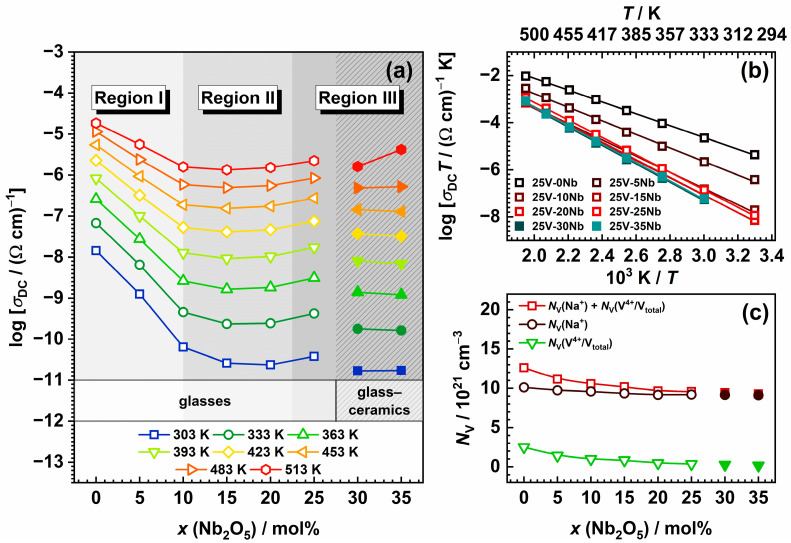
(**a**) Compositional and temperature dependence of DC conductivity, *σ*_DC_; (**b**) Arrhenius plot of DC conductivity; and (**c**) compositional dependence of number density, *N*_V_, of Na^+^ ions, V^4+^ ions, and the total number density of charge carriers. Empty symbols represent glasses, whereas full symbols denote glass–ceramics. The lines connecting data points in (**a**,**c**) are a guide for the eye, while the lines in (**b**) are obtained through linear regression.

Figure 6a illustrates the temperature-dependent variation in *σ*_DC_ for different Nb_2_O_5_ content, demonstrating that the DC conductivity is temperature-activated and exhibiting Arrhenius temperature dependence with characteristic activation energy, as shown in Figure 6b. The activation energy for DC conductivity, *E*_DC_, is calculated from the slope of the log(*σ*_DC_*T*) vs. 1000/*T*, utilising the Arrhenius equation (Equation (1)),

*σ*_DC_*T* = *σ*_0_*exp(−*E*_DC_/*k*_B_*T*), (1)

where *T* represents the temperature in K, *σ*_0_* is the pre-exponential factor, and *k*_B_ is the Boltzmann constant. The *σ*_DC_ at 303 K and the *E*_DC_ values for all samples are listed in Table 2, and they closely align with those reported for sodium phosphate-based glasses containing niobium [30,31] or vanadium [15,16] with compositions similar to those in this study.

Within the 25V series, both the DC conductivity and activation energy exhibit a non-monotonic trend. While *σ*_DC_ shows a decreasing trend with increasing Nb_2_O_5_ content, *E*_DC_ exhibits an opposite trend compared with *σ*_DC_, as anticipated. The highest *σ*_DC_ value corresponds to the starting glass, 25V-0Nb, and with the introduction of Nb_2_O_5_, it undergoes a reduction by approximately three orders of magnitude, reaching a minimum for the 25V-20Nb glass. This decrease is most pronounced for glasses with *x* ≤ 10, showing a drop in *σ*_DC_ of around 2.5 orders of magnitude. Beyond this point, *σ*_DC_ values exhibit less substantial changes. Similar observations apply to *E*_DC_, showing a progressive increase for *x* ≤ 10 and stabilization for 15 ≤ *x* ≤ 25; see Table 2. Furthermore, the *σ*_DC_ values for two glass–ceramic samples, 25V-30Nb and 25V-35Nb, exhibit a noticeable decrease when compared with those of glasses from the 25V series. This decrease in conductivity can be attributed to the significant presence of the Na_13_Nb_35_O_94_ phase in the two glass–ceramic samples, as its crystallization leads to the depletion of sodium ions within the residual glassy matrix. Moreover, the insufficient interconnection among grains of the Na_13_Nb_35_O_94_ crystal phase hinders the creation of an easy conductive pathway for the transport of Na^+^ ions, further contributing to the observed drop in conductivity.

Returning to the non-monotonic trends in *σ*_DC_ and *E*_DC_, it is noteworthy that the observed behaviour strikingly resembles the conductivity minimum seen in mixed ionic–polaronic systems containing both alkali and vanadium ions [15,16,17,18,19,20,21]. However, unlike these systems, where the gradual transition in the conduction mechanism from predominantly one mechanism (ionic–polaronic) to predominantly another is induced by changes in the amount of transition metal oxide and/or alkali metal oxide, in the 25V series, the quantities of both Na_2_O and V_2_O_5_ remain constant. This is reflected in the constant number density of the sodium ions, *N*_V_(Na^+^), calculated from the glass composition and density; see Table 2 and Figure 6c. Nonetheless, the number density of the polarons arising from the presence of reduced vanadium ions in the +4 oxidation state, *N*_V_(V^4+^/V_total_), calculated as the product of the number density of vanadium ions and the fraction of V^4+^ ions, is observed to gradually decrease with increasing Nb_2_O_5_ content, having a negative impact on the total number density of the charge carriers, *N*_V_(Na^+^) + *N*_V_(V^4+^). See Table 2 and Figure 6c. It is important to note that the main factor influencing the observed downward trend in the V^4+^/V_total_ ratio is determined through EPR measurements. As demonstrated in Table 2, there is a notable decrease in the fraction of V^4+^ ions with increasing Nb_2_O_5_ content. This observed downward trend aligns well with the existing literature [18,64,65] and can be influenced by synthesis parameters and the overall optical basicity of the glass. Given that increased temperatures and prolonged melting times result in a higher proportion of vanadium in the lower oxidation state, all melts in this study were uniformly held at the highest temperature for the same duration during synthesis. In addition to the synthesis parameters, the presence of V^4+^ ions is induced by the acidic environment created through a high P_2_O_5_ content, promoting the reduction of V^5+^ ions. On the other hand, the introduction of Nb_2_O_5_, known for its intrinsic basic nature and high electronic polarizability, results in a significant increase in basicity [34,61]. This, in turn, stabilizes a higher oxidation state of vanadium ions (V^5+^) [66], consequently leading to a notable decrease in the relative amount of the V^4+^ ions. See Table 2. Considering that the small polaron hopping mechanism is heavily dependent on the fraction of TM ions in a lower oxidation state [3,67], the decreasing trends observed in the fraction of V^4+^ (i.e., a decrease in the number of V^5+^–V^4+^ pairs) primarily contributes to the decreasing trend in *σ*_DC_. This serves as a significant indication that vanadium plays a key role in achieving the highest *σ*_DC_ values for 25V-0Nb and 25V-5Nb, with the subsequent decrease in conductivity stemming from the diminishing contribution of polaronic conductivity originating from the vanadium species in this mixed-conductive system. Specifically, the mixed-conduction mechanism in both the 25V-0Nb and 25V-5Nb glasses is evident in the markedly lower *E*_DC_ values of ~0.5 eV (see Table 2). These values are close to those of pure polaronic V-P glasses [68,69], indicating a significant polaronic contribution. Furthermore, utilising the method of internal friction, Barczyński and Murawski investigated the relaxation processes involving the migration of Na^+^ ions and the hopping of polarons between V^4+^ and V^5+^ ions in a Na-V-P glass system [17]. They established that the glass-sharing composition similar to that of the 25V-0Nb glass in this study displays one large, mixed electronic–ionic peak. This feature indicates a substantial presence of both mobile ions and polarons in the glass, reinforcing the observation that these glasses exhibit mixed conductivity. Nevertheless, with the gradual addition of Nb_2_O_5_, a progressive rise in *E*_DC_ is noted, reaching a steady value of ~0.7 eV (see Table 2), suggesting an emerging prominence of the ionic conductivity mechanism. This phenomenon is also evident in the previously discussed complex impedance spectra (see Figure 5b) where the 25V-0Nb and 25V-5Nb glasses (Region I), displaying mixed conductivity with a notable polaronic contribution, exhibit no noticeable effect of electrode polarization even at the elevated temperature of 513 K. In contrast, for samples where *x* ≥ 10 (Regions II and III), the “spur”, typical of ionically conductive materials, begins to emerge, suggesting an increasing influence of the ionic conductivity mechanism. All the aforementioned suggests that the addition of Nb_2_O_5_ triggers a transition in the conduction mechanism from mixed conductivity in Region I to predominantly ionic conductivity in Regions II and III. Furthermore, another essential factor influencing the observed decrease in polaronic contribution to the overall conductivity is the modification of the glass structure caused by the introduction of Nb_2_O_5_. Results from Raman and IR spectroscopy (see Figure 4) indeed indicate that within the 25V series, the introduction of Nb_2_O_5_ disrupts the structure of the initial, predominantly mixed V-P network, leading to its unravelling and the breakdown of the V–O–V bonds connecting the vanadate units. Considering that a low degree of crosslinking between vanadate structural units is recognized to hinder electron hopping along V^4+^–O–V^5+^ bonds [18,19,20], the introduction of Nb_2_O_5_, leading to the rupture of V–O–V bonds and an increased average distance between the vanadium ions, subsequently disrupts the pathways for electron hopping.

In the following section, the Summerfield scaling procedure is applied to conductivity spectra across a broad spectrum of frequencies and temperatures. This aims to explore the dynamics of charge carriers and acquire a more profound understanding of the electrical transport mechanism.

#### 2.4.3. Scaling Properties of Conductivity Spectra

The Summerfield scaling method is one of the most straightforward and widely adopted model-free scaling techniques, relying on two scaling parameters: the DC conductivity, *σ*_DC_, and the temperature, *T* [70]. It is mathematically represented by the equation (*σ*′(*ν*, *T*)/*σ*_DC_(*T*)) = F(*ν*/*σ*_DC_(*T*)*T*), where *σ*′ denotes the real component of conductivity and the other physical quantities maintain their conventional meanings. This method, recognized as mobility scaling, attains validity by affirming that temperature impacts charge carrier dynamics without modifying the conduction mechanism. Thus, the successful superposition of individual conductivity isotherms scaled by the common factor *σ*_DC_*T* in a double log–log plot of *σT* vs. *ν* indicates that the Summerfield scaling procedure is effective, signifying that the conduction mechanism remains unchanged with varying temperatures. On the contrary, the deviation from Summerfield scaling, as observed in mixed ion–polaron glasses with significant amounts of both ion and polaron charge carriers, is ascribed to differently thermally activated mobilities of ions and polarons [26].

**Figure 7:**
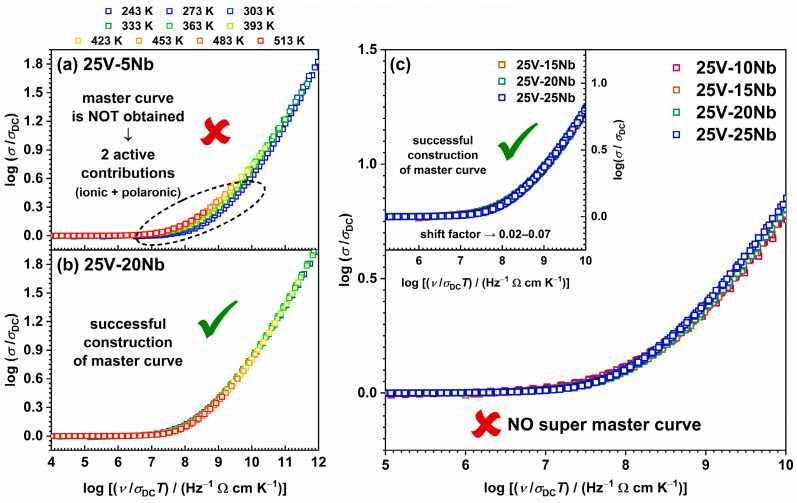
Summerfield scaling of conductivity spectra of (**a**) 25V-5Nb glass and (**b**) 25V-20Nb glass. (**c**) Construction of super master curve of the conductivity isotherms obtained by applying the Summerfield scaling procedure to all the investigated glasses; inset: individual master curves of glasses with 10 ≤ *x* ≤ 25 shifted along the x-axis to overlap with the reference master curve of 25V-10Nb glass.

The 25V-0Nb and 25V-5Nb glasses precisely exhibit this behaviour, with their conductivity spectra failing to overlap, as depicted in Figure 7a. Conversely, the described approach successfully generates perfect conductivity master curves for all glasses with *x* ≥ 10, as illustrated in Figure 7b. This result verifies the validity of time–temperature superposition (TTS), affirming the stability of the conduction mechanism regardless of temperature variations. The inability to scale the conductivity spectra of glasses with *x* < 10 aligns well with the previously mentioned findings, collectively suggesting that these glasses demonstrate mixed conductivity. On the other hand, the successful construction of master curves for glasses where *x* ≥ 10 additionally confirms that the incorporation of Nb_2_O_5_ leads to the prevalence of the ionic conduction mechanism in these glasses.

An additional validation of the Summerfield scaling results can be attained through the following approach. Specifically, this scaling method is considered to be satisfied if the slope of the line log(*σ*’*T*) vs. log*ν*_0_, where *ν*_0_ represents the onset frequency of conductivity dispersion defined by the equation *σ*’(*ν*_0_) = 2*σ*_DC_, is equal to 1. Indeed, all glasses for which master curves are successfully constructed adhere to this criterion, as illustrated in Figure S2 in the Supplementary Materials. On the other hand, for the glass samples 25V-0Nb and 25V-5Nb, the observed deviation from a slope of 1 aligns with expectations, given that Summerfield scaling fails to produce master curves.

In the next step, individual conductivity master curves are superimposed to investigate the influence of glass composition and structure on conductivity dispersion. It should be noted that two glass–ceramic samples, 25V-30Nb and 25V-35Nb, are excluded from the analysis due to partial crystallization. Additionally, two glasses—specifically, 25V-0Nb and 25V-5Nb—are omitted from the analysis as well, because they do not comply with the scaling criteria of the Summerfield procedure. As depicted in Figure 7c, the attempt to construct a super master curve for glasses with 10 ≤ *x* ≤ 25 from the 25V series proves unsuccessful. Instead, the master curve for the 25V-10Nb glass displays a distinct shape compared with the other three glasses, all of which share the same shape. While the master curves of the 25V-15Nb and 25V-20Nb glasses perfectly overlap, the master curve for the 25V-25Nb glass exhibits a slight shift toward lower values of log(*σ*_DC_*T*). Nevertheless, it aligns well with the other two master curves when shifted along the scaled frequency axis; see the inset of Figure 7c. Here, it is worth noting the correlation between the scaling outcomes for the investigated glasses and their *σ*_DC_ and *E*_DC_ values. Specifically, the shape of the master curves is observed to change concurrently with the changes in *σ*_DC_, mirroring phenomena observed in our previous study on another series within the Na-V-P-Nb system [27]. Furthermore, these alterations in the master curves coincide with the transition of the conductivity mechanism from mixed conductive in glasses with *x* < 10 to predominantly ionic in glasses with *x* ≥ 10. In the case of the initial two glasses from the 25V series, 25V-0Nb and 25V-5Nb, this is evidenced by the inability to obtain master curves because of their mixed ion–polaron conductivity. Furthermore, for the 25V-10Nb glass, which exhibits the largest drop in *σ*_DC_ compared with the initial glass and marks the composition of significant structural modifications induced by Nb_2_O_5_ introduction, the master curve assumes a distinctive shape differing from the other successfully constructed master curves. On the other hand, the 25V-15Nb, 25V-20Nb, and 25V-25Nb glasses, exhibiting successful super master construction, also show similar *σ*_DC_ and *E*_DC_ (see Table 2), indicating that they possess similar local structural environments for Na^+^ transport. Based on all of the aforementioned observations, the following conclusions can be drawn: (i) the extent of the mixed ion–polaron conduction mechanism is strongest for 25V-0Nb and 25V-5Nb glasses, indicated by the inability to construct their master curves; (ii) the successful construction of a master curve for 25V-10Nb implies a diminishing contribution of polaronic conductivity and an increasing influence of ionic conductivity as the predominant mechanism of electrical conductivity; and (iii) the 25V-15Nb, 25V-20Nb, and 25V-25Nb glasses exhibit predominantly ionic conduction mechanisms and possess similar local structural environments for Na^+^ transport, as evidenced by the same-shaped master curves.

### 2.5. Influence of V_2_O_5_ Content on the Mechanism of Electrical Conductivity

As consistently detailed throughout the text, the 25V series distinguishes itself with unique behaviour, differentiating it from other alkali–niobate–phosphate (A-Nb-P) systems where P_2_O_5_ is gradually replaced with Nb_2_O_5_ [27,28,29,30,31,32,33,34]. This distinct effect is evident not only in its general and thermal properties but also in its electrical characteristics. Specifically, *V*_M_, *T*_g_, and *σ*_DC_ exhibit non-monotonic trends resulting from structural changes induced by the addition of Nb_2_O_5_. While *T*_g_ and *σ*_DC_ show a decreasing trend, reaching their minimum values in the 25V-20Nb glass, *V*_M_ follows a positive trend, reaching its peak with the same glass composition. It is intriguing because the addition of Nb_2_O_5_ typically has a beneficial impact on the thermal properties of alkali-phosphate-based systems manifested by an increase in *T*_g_ and a reduction in *V*_M_, indicative of the formation of a more compact mixed Nb-P network [27,29,30,31,32,33,34]. Additionally, the incorporation of Nb_2_O_5_ is usually recognized for its ability to enhance electrical conductivity in alkali-phosphate-based systems by forming mixed P–O–Nb bonds, thereby facilitating the transport of alkali ions through the glass network [28]. However, the 25V series deviates from this behaviour, showing an unfavourable impact of Nb_2_O_5_ on both thermal and electrical properties. What sets the 25V series apart from other A-Nb-P systems is the notable presence of a relatively high quantity of V_2_O_5_ (25 mol%), which may directly account for the distinctive trends observed in this series.

To shed light on the influence of V_2_O_5_ content on the observed trends arising from the gradual substitution of P_2_O_5_ with Nb_2_O_5_ in this series, a valuable comparison can be made between the 25V series and the outcomes reported in the previously published 35Na_2_O-10V_2_O_5_-(55 − *x*)P_2_O_5_ − *x*Nb_2_O_5_ (10V) series [27]. Both of these systems consist of the same oxides and maintain constant proportions of Na_2_O (35 mol%) and V_2_O_5_ (10/25 mol%), with P_2_O_5_ being replaced by Nb_2_O_5_. Hence, changing the concentration of V_2_O_5_ content allows for the assessment of the extent of its influence on the properties under examination. A comprehensive exploration of the general, thermal, structural, and electrical properties of the 10V series in Ref. [27] reveals that this series exhibits trends consistent with the majority of the A-Nb-P systems documented in the literature [27,28,29,30,31,32,33,34]. In this series, V_2_O_5_ is recognized to play dual roles within the structure, acting as both a glass modifier and a network former; nevertheless, owing to its relatively small quantity, it exerts no substantial influence on the overall trends, unlike in the 25V series. Indeed, the latter series exhibits behaviour that is entirely contrasting to that of the 10V series, as illustrated in the following.

The addition of Nb_2_O_5_ has different effects on the change in *V*_M_ in two series, attributable to structural modifications induced by its incorporation. In series 10V, the addition of Nb_2_O_5_ leads to a more compact structure, reflected in the downward trend of *V*_M_ [27]. This is further substantiated by the vibrational spectroscopy results, unveiling a structural evolution from predominantly phosphate (Region I) through predominantly mixed Nb-P (Region II) to a predominantly niobate network (Region III). Conversely, in the 25V series, the incorporation of Nb_2_O_5_ has an opposing effect, disrupting the initially compact mixed V-P network, which leads to a more “open” network, as evidenced by the increasing *V*_M_ values. Interestingly, the Raman/IR-ATR spectroscopy finding reveals some similarities between the 10V and 25V series; nevertheless, notable distinctions exist, with the most significant being that in the 25V series, Region I corresponds to a predominantly mixed V-P network. As the Nb_2_O_5_ content increases, the signals describing NbO_6_ units connected into chains via Nb–O–Nb bridging bonds progressively dominate (Region II). At the highest Nb_2_O_5_ content, these units tend to cluster, forming a 3D network in Region III. Due to the lower initial amount of P_2_O_5_ (a consequence of a higher V_2_O_5_ content), the GFR in the 25V series is narrower compared with that in the 10V series. For the same reason, introducing Nb_2_O_5_ into the initially mixed V-P network in the 25V series triggers the formation of 3D clusters at a lower Nb_2_O_5_ concentration (25 mol%), in contrast to the 40 mol% Nb_2_O_5_ concentration required for niobate clustering to dominate in the 10V series.

**Figure 8:**
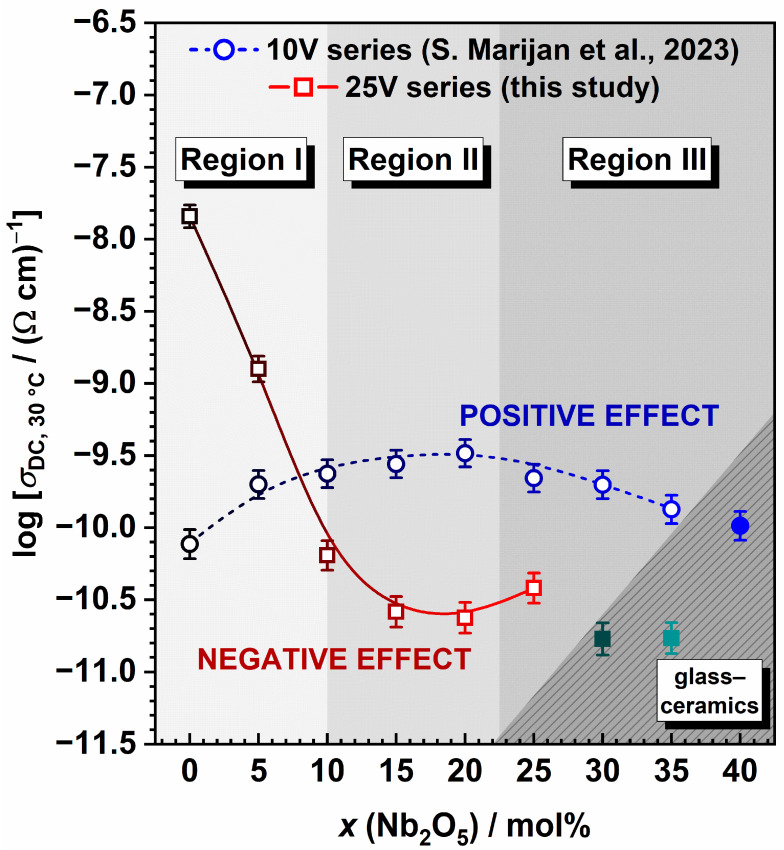
Comparison of compositional dependence of DC conductivity, *σ*_DC_, at 303 K for two glass and glass-ceramics series, 35Na_2_O-10V_2_O_5_-(55 − *x*)P_2_O_5_ − *x*Nb_2_O_5_ [27] and 35Na_2_O-25V_2_O_5_-(40 − *x*)P_2_O_5_ − *x*Nb_2_O_5_. The lines connecting data points are a guide for the eye.

The electrical transport mechanism is also found to be markedly affected by the V_2_O_5_ content, and comparing the two series enables assessing the extent of its polaronic contribution. Specifically, an in-depth examination of the electrical properties in the 10V series reveals that V_2_O_5_, at a concentration of 10 mol%, does not actively participate in the overall conduction process through polaronic transport. The mechanism of electrical conductivity in the 10V series is identified as purely ionic, and the observed non-monotonic trends in *σ*_DC_ and *E*_DC_ are ascribed to the facilitating effect of Nb_2_O_5_ on the transport of Na^+^ ions, resulting in the positive mixed glass former effect [27]. See Figure 8. On the contrary, the 25V series displays an opposite trend, wherein the substitution of P_2_O_5_ by Nb_2_O_5_ decreases *σ*_DC_ (see Figure 8). This observation is intricately tied to the substantial amounts of two types of charge carriers, ionic (35 mol% Na_2_O) and, especially, polaronic (25 mol% V_2_O_5_), leading to mixed conductivity in the 25V-0Nb and 25V-5Nb glasses. With the addition of more Nb_2_O_5_, the polaronic contribution diminishes as the number of V^5+^–V^4+^ pairs decreases and the mixed V-P network, crucial for enabling favourable electron transfer pathways, undergoes disruption and transitions to a mixed Nb-V-P network.

**Figure 9:**
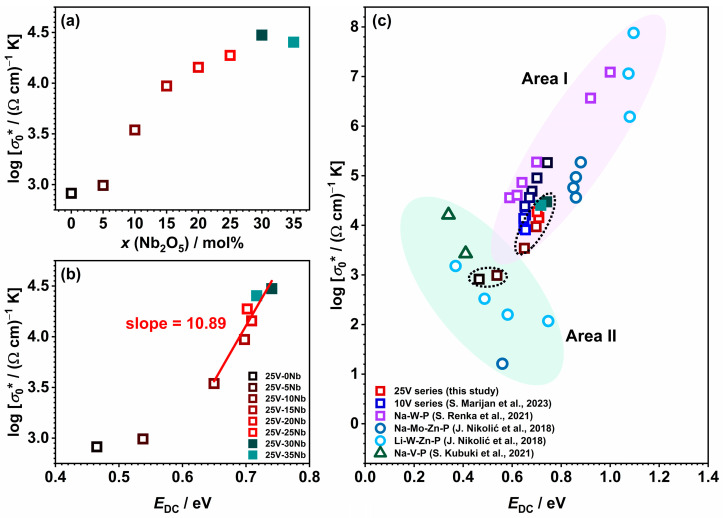
The dependence of the pre-exponential factor (*σ*_0_*) as a function of (**a**) composition and (**b**) activation energy (*E*_DC_); and (**c**) a comparison of the glasses and glass–ceramics from this study with those from 10V series [27] and systems Na-W-P [22], Na-Mo-Zn-P [25], Li-W-Zn-P [25] and Na-V-P [23] from the literature. The line in (**b**) is obtained through linear regression. The dotted circles in Figure 9c highlight the samples from this study.

An effective approach to visualize the complexities of mixed conductive glass systems like the 25V series involves employing the Meyer–Neldel (M–N) formalism [71]. According to this rule, the pre-exponential factor *σ*_0_* can be linked to *E*_DC_, using the equation log *σ*_0_* = a *E*_DC_ + b, where a and b are constants. A negative slope indicates electronic conductivity, while a positive slope suggests ionic conductivity. This allows for distinguishing various conduction mechanisms, as seen in studies across different materials, including glasses with ionic, electronic, or mixed electronic–ionic conduction [24]. When examining the pre-exponential factor, *σ*_0_*, in relation to composition (see Figure 9a) and *E*_DC_ (see Figure 9b), a positive relationship is observed for samples with 10 ≤ *x* ≤ 35, indicating predominant ionic electrical transport. However, glasses with *x* < 10, displaying a noteworthy contribution of polaronic conductivity, deviate from this trend and exhibit lower values for both *σ*_0_* and *E*_DC_. A comparison with different glass series, including the 10V series—showcasing ionic [22,27], electronic [23], or mixed electronic–ionic conduction [24,25,26]—reveals that the glasses–(ceramics) with 10 ≤ *x* ≤ 35 fall within Area I, signifying glasses with dominant ionic conductivity (see Figure 9c). On the other hand, 25V-0Nb glass and 25V-5Nb are positioned in Area II, corresponding to glasses with a dominant polaronic conduction mechanism.

## 3. Materials and Methods

Glass samples within the quaternary system 35Na_2_O-25V_2_O_5_-(40 − *x*)P_2_O_5_ − *x*Nb_2_O_5_ (25V), *x* = 0–35, mol%, are synthesized using the conventional melt quenching technique in 3 g batches. The precursors, Na_2_CO_3_, NH_4_H_2_PO_4_, Nb_2_O_5_, and V_2_O_5_, are precisely weighed and mixed in the desired stoichiometric proportions. Homogenization is achieved through 15 min of grinding in an agate mortar. The prepared reaction mixtures undergo calcination at 700 °C, followed by melting in a platinum crucible under ambient air within the temperature range of 1100 to 1200 °C. After a 40 min hold time at a specific temperature, black and opaque products are obtained by pouring them into a stainless-steel mould at RT. Given a weight loss of less than 1.5%, the composition of the glass batch is considered an accurate representation of the actual glass composition. The samples are designated according to the mol% of V_2_O_5_ and Nb_2_O_5_ in the batch. For example, the glass labelled as 25V-20Nb comprises 25 mol% V_2_O_5_ and 20 mol% Nb_2_O_5_, as outlined in Table 1.

The determination of sample density, *ρ*, for bulk samples is conducted at RT using Archimedes’ method, with 96% ethanol employed as the immersion liquid. The molar volume, *V*_M_, is calculated as *V*_M_ = *M*/*ρ*, where *M* represents the average molar weight of the glass.

The thermal characteristics of the prepared samples are investigated using differential thermal analysis (DTA) utilising the Mettler TGA/DSC 3+ thermobalance. Powder samples (~30 mg) are placed in a Pt crucible in an oxygen atmosphere, with a heating rate of 20 °C min^−1^ over the temperature range of 25–1000 °C. The results are analysed using the Mettler STARe 9.01 software, and the glass transition temperature (*T*_g_) is determined for all the samples along with the first observable crystallization peak temperature (*T*_c_).

Powder X-ray diffraction (PXRD) data are obtained using a Bruker D8 Discover diffractometer equipped with a LYNXEYE XE-T detector (Bruker AXS GmbH, Karlsruhe, Germany). Data are recorded in Bragg–Brentano geometry in the 2*θ* range of 10° to 70° using CuKα radiation (1.5418 Å). Rietveld analysis of the diffraction patterns is performed with the HighScore X’pert HighScore Plus 3.0 program (Malvern Panalytical, Almelo, The Netherlands). The amorphous structure of the glasses is confirmed, and the partially crystallized samples with high Nb_2_O_5_ content (25V-30Nb and 25V-35Nb) are analysed both qualitatively and quantitatively. The quantitative analysis employs the internal standard method, using crystalline ZnO as an internal standard, with weight fractions of the amorphous and crystalline phases determined with Rietveld refinement [72]. In a system with a known amount of standard S, the weight fraction of the crystalline phase P was determined according to the following equation (Equation (2)):

*W*_P_ = [*W*_P_*S*_P_(*ZMV*)_P_]/[*S*_S_(*ZMV*)_S_] × [1/(1 − *W*_S_)], (2)

where *S* is the scale factor, *Z* is the number of the formula unit within the unit cell, *M* is the formula unit mass, and *V* is the volume of the unit cell. The weight fraction of the amorphous phase was determined by the following expression (Equation (3)):

*W*_A_ = 1 − *W*_S_ − Σ*W*_P_, (3)

where *W*_S_, *W*_P_, and *W*_A_ are the weight percentages of the standard, crystalline, and amorphous phases, respectively.

Raman spectra are measured from bulk samples at RT across the spectral range of 1500–60 cm^−1^ using a Thermo Scientific DXR Raman spectrometer with a 532 nm solid-state (Nd: YAG) diode-pumped laser. The acquired Raman spectra exhibit a complex shape, analysed using a least-square fitting procedure, assuming a Gaussian shape for all bands. From the deconvoluted Raman spectra, the position and intensity of individual components are determined. The Q*^n^* notation, denoting the number of bridging oxygen atoms per PO_4_ tetrahedron (*n* = 0–3), is used to represent phosphate units. Additionally, attenuated total reflectance infrared (IR-ATR) spectra of powder samples are recorded using a Perkin Elmer Spectrum Two FT-IR Spectrometer equipped with a diamond universal attenuated total reflectance (UATR) accessory within a spectral range of 4000–400 cm^−1^.

Electron paramagnetic resonance (EPR) spectra of bulk samples are acquired at RT utilising a cw-ESR EMXmicro spectrometer (Bruker), functioning in the X band (~9.83 GHz) and equipped with Xenon software. Sample weights are determined to the nearest hundredth of a milligram and the obtained EPR spectra are double-integrated, while experimental parameters are employed to normalize the areas of all spectra. Utilising Mn^2+^ as a standard, the spin concentration is determined as spin/g for all samples, and the V^4+^/V_total_ ratio is calculated from the obtained data.

The Axia™ ChemiSEM™ Scanning Electron Microscope (Thermo Fisher Scientific, Waltham, MA, USA) with an energy-dispersive X-ray spectroscopy (EDS) system is employed to analyse the microstructure and elemental composition of the prepared samples.

Solid-state impedance spectroscopy (SS-IS) is utilised for electrical measurements. The annealed samples are crafted into ~1 mm thick disks with gold electrodes (5.4 mm in diameter) sputtered on both sides using a Sputter Coater SC7620. Electrical properties are determined by measuring complex impedance with a Novocontrol Alpha-AN Dielectric Spectrometer (Novocontrol Technologies GmbH & Co., KG, Hundsangen, Germany) across a wide range of frequencies (0.01 Hz–1 MHz) and temperatures (183 K to 513 K), maintaining a temperature control accuracy of ±0.2 K. The obtained impedance spectra are analysed through modelling with the appropriate electrical equivalent circuit (EEC) using the complex non-linear least-squares (CNLLSQ) method and WinFIT software (version 3.2, Novocontrol Technologies GmbH & Co., KG, Hundsangen, Germany). Impedance spectra displaying a depressed semicircle are fitted using the EEC model, which includes a parallel combination of a resistor (R) and a constant phase element (CPE). In cases where impedance spectra exhibit an additional low-frequency “spur” attributed to the electrode polarization effect, the corresponding EEC model integrates a parallel R-CPE combination connected in series with another CPE.

## 4. Conclusions

This study investigates the influence of introducing two transition metal oxides, V_2_O_5_ and Nb_2_O_5_, on the thermal, (micro)structural, and electrical properties of a mixed conductive glass and glass–ceramic system with the nominal composition 35Na_2_O-25V_2_O_5_-(40 − *x*)P_2_O_5_ − *x*Nb_2_O_5_ (*x* = 0–35, mol%). Solid-state impedance spectroscopy measurements and differential thermal analysis indicate that the initial glass is the optimal sample, exhibiting the highest values for both direct current (DC) conductivity and glass transition temperature. Our findings highlight that the gradual substitution of P_2_O_5_ with Nb_2_O_5_ impacts both DC conductivity and glass transition temperature, resulting in non-linear trends, with the lowest values observed for the glass containing 20 mol% Nb_2_O_5_. An extensive analysis of the electrical properties, coupled with electron paramagnetic resonance measurements and vibrational spectroscopy results, indicates that the observed non-monotonic trend in DC conductivity arises from the transition of the electrical conductivity mechanism from mixed conductive to predominantly ionic. This is associated with a decrease in the contribution of polaronic conductivity due to two factors: the reduction in the relative number of V^4+^ ions and the structural modifications of the glass network resulting from the introduction of Nb_2_O_5_. While the former results in a reduction in the V^5+^–V^4+^ pairs available for participation in the small polaron hopping mechanism, the latter disrupts the predominantly mixed vanadate–phosphate network in the initial glasses, causing the breakage of the V–O–V bonds and an increased average distance between vanadium ions. This ultimately impedes electron hopping pathways and hinders polaronic transport. The mechanism of electrical transport is additionally examined through the model-free Summerfield scaling procedure, revealing that glasses characterized by a predominantly mixed vanadate–phosphate network (*x* < 10 mol%, Region I) display mixed conductivity. Meanwhile, glasses featuring a mixed Nb-V-P glass network (10 mol% ≤ *x* ≤ 20 mol%, Region II) and a predominantly mixed Nb-V network (*x* ≥ 25 mol%, Region III) reveal the dominance of the ionic conductivity mechanism. Furthermore, a comparative analysis of two analogue series with varying V_2_O_5_ content (10 mol% and 25 mol%) is conducted to assess the impact of V_2_O_5_ content on the electrical transport mechanism and to evaluate the extent of its polaronic contribution. We illustrate that at 10 mol%, V_2_O_5_ does not actively partake in the overall conduction process through polaronic transport, given its low concentration. However, elevating the V_2_O_5_ content to 25 mol% leads to a substantial enhancement in DC conductivity (a jump by more than two orders of magnitude), attributed to the significant contribution of V_2_O_5_ to the total conductivity via polaron transport. The results of this study offer valuable insights into the mixed conductive glass and glass–ceramic Na-V-P-Nb-based systems. They showcase the capability to modify the mechanism of electrical conductivity through straightforward adjustments in composition, a crucial aspect in the design and development of novel cathode materials for sodium-ion batteries composed of glassy and glass–ceramic materials.

## Figures and Tables

**Table 1:** Batch composition and selected properties for all glass and glass–ceramic samples.

Sample	*x* (mol%)				O/P	*Ρ* (g cm^−3^)	*V*_M_ (cm^3^ mol^−1^)	*T*_g_ (°C)	*T*_c_ (°C)	*T*_c_*− T*_g_ (°C)
	Na_2_O	V_2_O_5_	P_2_O_5_	Nb_2_O_5_						
25V-0Nb	35	25	40	0	4.50	2.97	41.69	371	450	79
25V-5Nb	35	25	35	5	5.14	3.00	43.31	348	439	91
25V-10Nb	35	25	30	10	6.00	3.10	43.94	340	434	94
25V-15Nb	35	25	25	15	7.20	3.16	45.12	334	444	110
25V-20Nb	35	25	20	20	9.00	3.23	46.00	338	450	112
25V-25Nb	35	25	15	25	12.00	3.38	45.78	355	468	113
25V-30Nb	35	25	10	30	-	3.50	-	230	357	127
25V-35Nb	35	25	5	35	-	3.61	-	201	284	83

**Table 2:** DC conductivity, *σ*_DC_; activation energy, *E*_DC_; pre-exponential factor, *σ*_0_*; number densities of sodium ions, *N*_V_ (Na^+^), and vanadium ions, *N*_V_ (V^4+^); and the relative amount of V^4+^.

Sample	*σ*_DC_ at 303 K (Ω^−1^ cm^−1^)	*E*_DC_ (eV)	log *σ*_0_* (Ω^−1^ cm^−1^ K)	*N*_v_ (Na^+^) (10^21^ cm^−3^)	*N*_v_ (V^4+^/V_total_) (10^21^ cm^−3^)	V^4+^/V_total_ (%)
25V-0Nb	1.44 × 10^−8^	0.47	2.91	10.1	2.5	34.7
25V-5Nb	1.26 × 10^−9^	0.57	2.99	9.7	1.4	20.5
25V-10Nb	6.43 × 10^−11^	0.68	3.54	9.6	1.0	14.4
25V-15Nb	2.61 × 10^−11^	0.73	3.97	9.3	0.8	12.6
25V-20Nb	2.37 × 10^−11^	0.74	4.16	9.2	0.5	7.4
25V-25Nb	3.81 × 10^−11^	0.74	4.27	9.2	0.4	5.6
25V-30Nb	1.69 × 10^−11^	0.78	4.47	9.2	0.3	4.3
25V-35Nb	1.72 × 10^−11^	0.77	4.40	9.1	0.2	2.7

## Data Availability

The data presented in this study are available from the corresponding author upon request.

## Supplementary Materials

The following supporting information can be downloaded at: https://www.mdpi.com/article/10.3390/ijms25053005/s1.

## References

[1] Singh: A.N., Islam M., Meena A., Faizan M., Han D., Bathula C., Hajibabaei A., Anand R., Nam K.-W. (2023). Unleashing the Potential of Sodium-Ion Batteries: Current State and Future Directions for Sustainable Energy Storage. Adv. Funct. Mater..

[2] Zhao C., Liu L., Qi X., Lu Y., Wu F., Zhao J., Yu Y., Hu Y.-S., Chen L. (2018). Solid-State Sodium Batteries. Adv. Energy Mater..

[3] Austin I.G., Mott N.F. (1969). Polarons in Crystalline and Non-Crystalline Materials. Adv. Phys..

[4] Thirupathi R., Kumari V., Chakrabarty S., Omar S. (2023). Recent Progress and Prospects of NASICON Framework Electrodes for Na-Ion Batteries. Prog. Mater. Sci..

[5] Zhou Q., Wang L., Li W., Zhao K., Liu M., Wu Q., Yang Y., He G., Parkin I.P., Shearing P.R. (2021). Sodium Superionic Conductors (NASICONs) as Cathode Materials for Sodium-Ion Batteries. Electrochem. Energy Rev..

[6] Novikova S.A., Larkovich R.V., Chekannikov A.A., Kulova T.L., Skundin A.M., Yaroslavtsev A.B. (2018). Electrical Conductivity and Electrochemical Characteristics of Na_3_V_2_(PO_4_)_3_-Based NASICON-Type Materials. Inorg. Mater.

[7] Zheng W., Gao R., Zhou T., Huang X. (2018). Enhanced Electrochemical Performance of Na_3_V_2_(PO_4_)_3_ with Ni^2+^ Doping by a Spray Drying-Assisted Process for Sodium Ion Batteries. Solid State Ion..

[8] Liu X., Feng G., Wu Z., Yang Z., Yang S., Guo X., Zhang S., Xu X., Zhong B., Yamauchi Y. (2020). Enhanced Sodium Storage Property of Sodium Vanadium Phosphate via Simultaneous Carbon Coating and Nb^5+^ Doping. Chem. Eng. J..

[9] Bi L., Liu X., Li X., Chen B., Zheng Q., Xie F., Huo Y., Lin D. (2020). Modulation of the Crystal Structure and Ultralong Life Span of a Na_3_V_2_(PO_4_)_3_-Based Cathode for a High-Performance Sodium-Ion Battery by Niobium–Vanadium Substitution. Ind. Eng. Chem. Res..

[10] Rao X., Wang J., Yang M.-A., Zhao H., Li Z. (2022). A Superior Na_3_V_2_(PO_4_)_3_-Based Cathode Enhanced by Nb-Doping for High-Performance Sodium-Ion Battery. APL Mater..

[11] Li X., Huang Y., Wang J., Miao L., Li Y., Liu Y., Qiu Y., Fang C., Han J., Huang Y. (2018). High Valence Mo-Doped Na_3_V_2_(PO_4_)_3_/C as a High Rate and Stable Cycle-Life Cathode for Sodium Battery. J. Mater. Chem. A.

[12] Sun S., Chen Y., Cheng J., Tian Z., Wang C., Wu G., Liu C., Wang Y., Guo L. (2021). Constructing Dimensional Gradient Structure of Na_3_V_2_(PO_4_)_3_/C@CNTs-WC by Wolfram Substitution for Superior Sodium Storage. Chem. Eng. J..

[13] Gandi S., Chidambara Swamy Vaddadi V.S., Sripada Panda S.S., Goona N.K., Parne S.R., Lakavat M., Bhaumik A. (2022). Recent Progress in the Development of Glass and Glass-Ceramic Cathode/Solid Electrolyte Materials for next-Generation High Capacity All-Solid-State Sodium-Ion Batteries: A Review. J. Power Sources.

[14] Wang Z., Luo S., Zhang X., Guo S., Li P., Yan S. (2023). Glass and Glass Ceramic Electrodes and Solid Electrolyte Materials for Lithium Ion Batteries: A Review. J. Non-Cryst. Solids.

[15] Wasiucionek M., Garbarczyk J., Kurek P., Jakubowski W. (1994). Electrical Properties of Glasses of the Na_2_O-V_2_O_5_-P_2_O_5_ System. Solid State Ion..

[16] Ungureanu M.C., Lévy M., Souquet J.L. (1998). Mixed Conductivity of Glasses in the P_2_O_5_-V_2_O_5_-Na_2_O System. Ionics.

[17] Barczyński R.J., Murawski L. (2006). Mixed Electronic-Ionic Conductivity in Vanadate Oxide Glasses Containing Alkaline Ions. Mater. Sci. Pol..

[18] Barczyński R.J., Król P., Murawski L. (2010). Ac and Dc Conductivities in V_2_O_5_–P_2_O_5_ Glasses Containing Alkaline Ions. J. Non-Cryst. Solids.

[19] Garbarczyk J.E., Wasiucionek M., Jóźwiak P., Tykarski L., Nowiński J.L. (2002). Studies of Li_2_O–V_2_O_5_–P_2_O_5_ Glasses by DSC, EPR and Impedance Spectroscopy. Solid State Ion..

[20] Jozwiak P., Garbarczyk J.E. (2005). Mixed Electronic–Ionic Conductivity in the Glasses of the Li_2_O–V_2_O_5_–P_2_O_5_ System. Solid State Ion..

[21] Takahashi H., Karasawa T., Sakuma T., Garbarczyk J.E. (2010). Electrical Conduction in the Vitreous and Crystallized Li_2_O–V_2_O_5_–P_2_O_5_ System. Solid State Ion..

[22] Renka S., Pavić L., Tricot G., Mošner P., Koudelka L., Moguš-Milanković A., Šantić A. (2021). A Significant Enhancement of Sodium Ion Conductivity in Phosphate Glasses by Addition of WO_3_ and MoO_3_: The Effect of Mixed Conventional–Conditional Glass-Forming Oxides. Phys. Chem. Chem. Phys..

[23] Kubuki S., Osouda K., Ali A.S., Khan I., Zhang B., Kitajou A., Okada S., Okabayashi J., Homonnay Z., Kuzmann E. (2021). 57Fe-Mössbauer and XAFS Studies of Conductive Sodium Phospho-Vanadate Glass as a Cathode Active Material for Na-Ion Batteries with Large Capacity. J. Non-Cryst. Solids.

[24] Pavić L., Šantić A., Nikolić J., Mošner P., Koudelka L., Pajić D., Moguš-Milanković A. (2018). Nature of Mixed Electrical Transport in Ag_2_O–ZnO–P_2_O_5_ Glasses Containing WO_3_ and MoO_3_. Electrochim. Acta.

[25] Nikolić J., Pavić L., Šantić A., Mošner P., Koudelka L., Pajić D., Moguš-Milanković A. (2018). Novel Insights into Electrical Transport Mechanism in Ionic-Polaronic Glasses. J. Am. Ceram. Soc..

[26] Šantić A., Nikolić J., Pavić L., Banhatti R.D., Mošner P., Koudelka L., Moguš-Milanković A. (2019). Scaling Features of Conductivity Spectra Reveal Complexities in Ionic, Polaronic and Mixed Ionic-Polaronic Conduction in Phosphate Glasses. Acta Mater..

[27] Marijan S., Razum M., Klaser T., Mošner P., Koudelka L., Skoko Ž., Pisk J., Pavić L. (2023). Tailoring Structure for Improved Sodium Mobility and Electrical Properties in V_2_O_5_–Nb_2_O_5_–P_2_O_5_ Glass(Es)-(Ceramics). J. Phys. Chem. Solids.

[28] Chowdari B.V.R., Radhakrishnan K. (1989). Electrical and Electrochemical Characterization of Li_2_O:P_2_O_5_:Nb_2_O_5_-Based Solid Electrolytes. J. Non-Cryst. Solids.

[29] Flambard A., Videau J.J., Delevoye L., Cardinal T., Labrugère C., Rivero C.A., Couzi M., Montagne L. (2008). Structure and Nonlinear Optical Properties of Sodium–Niobium Phosphate Glasses. J. Non-Cryst. Solids.

[30] Honma T., Okamoto M., Togashi T., Ito N., Shinozaki K., Komatsu T. (2015). Electrical Conductivity of Na_2_O–Nb_2_O_5_–P_2_O_5_ Glass and Fabrication of Glass–Ceramic Composites with NASICON Type Na_3_Zr_2_Si_2_PO_12_. Solid State Ion..

[31] Benyounoussy S., Bih L., Muñoz F., Rubio-Marcos F., Naji M., El Bouari A. (2021). Structure, Dielectric, and Energy Storage Behaviors of the Lossy Glass-Ceramics Obtained from Na_2_O-Nb_2_O_5_-P_2_O_5_ Glassy-System. Phase Transit..

[32] Senapati A., Barik S.K., Venkata Krishnan R., Chakraborty S., Jena H. (2023). Studies on Synthesis, Structural and Thermal Properties of Sodium Niobium Phosphate Glasses for Nuclear Waste Immobilization Applications. J. Therm. Anal. Calorim..

[33] Mošner P., Hostinský T., Koudelka L. (2022). Thermal, Structural and Crystallization Study of Na_2_O–P_2_O_5_–Nb_2_O_5_ Glasses. J. Solid State Chem..

[34] Koudelka L., Kalenda P., Mošner P., Montagne L., Revel B. (2021). Potassium Niobato-Phosphate Glasses and Glass-Ceramics. J. Non-Cryst. Solids.

[35] Lide D.R. (2005). Bond Strengths in Diatomic Molecules. CRC Handbook of Chemistry and Physics.

[36] Bih L., Azrour M., Manoun B., Graça M.P.F., Valente M.A. (2013). Raman Spectroscopy, X-Ray, SEM, and DTA Analysis of Alkali-Phosphate Glasses Containing WO_3_ and Nb_2_O_5_. J. Spectrosc..

[37] Zheng Q., Zhang Y., Montazerian M., Gulbiten O., Mauro J.C., Zanotto E.D., Yue Y. (2019). Understanding Glass through Differential Scanning Calorimetry. Chem. Rev..

[38] Craig D.C., Stephenson N.C. (1971). The Structure of the Bronze Na_13_Nb_35_O_94_ and the Geometry of Ferroelectric Domains. J. Solid State Chem..

[39] Benyounoussy S., Bih L., Muñoz F., Rubio-Marcos F., EL Bouari A. (2021). Effect of the Na_2_O–Nb_2_O_5_–P_2_O_5_ Glass Additive on the Structure, Dielectric and Energy Storage Performances of Sodium Niobate Ceramics. Heliyon.

[40] Razum M., Pavić L., Ghussn L., Moguš-Milanković A., Šantić A. (2021). Transport of Potassium Ions in Niobium Phosphate Glasses. J. Am. Ceram. Soc..

[41] Rambo C.R., Ghussn L., Sene F.F., Martinelli J.R. (2006). Manufacturing of Porous Niobium Phosphate Glasses. J. Non-Cryst. Solids.

[42] Attafi Y., Liu S. (2016). Conductivity and Dielectric Properties of Na_2_O-K_2_O-Nb_2_O_5_-P_2_O_5_ Glasses with Varying Amounts of Nb_2_O_5_. J. Non-Cryst. Solids.

[43] Wang B., Greenblatt M., Yan J. (1994). Ionic Conductivities of Crystalline and Glassy Na_4_NbP_3_O_12_ and Crystalline Na_6_Nb_2_P_6_O_23_. Solid State Ionics.

[44] Chu C.M., Wu J.J., Yung S.W., Chin T.S., Zhang T., Wu F.B. (2011). Optical and Structural Properties of Sr–Nb–Phosphate Glasses. J. Non-Cryst. Solids.

[45] Iordanova R., Milanova M., Aleksandrov L., Shinozaki K., Komatsu T. (2020). Structural Study of WO_3_-La_2_O_3_-B_2_O_3_-Nb_2_O_5_ Glasses. J. Non-Cryst. Solids.

[46] Karam L., Adamietz F., Rodriguez V., Bondu F., Lepicard A., Cardinal T., Fargin E., Richardson K., Dussauze M. (2020). The Effect of the Sodium Content on the Structure and the Optical Properties of Thermally Poled Sodium and Niobium Borophosphate Glasses. J. Appl. Phys..

[47] Teixeira Z., Alves O.L., Mazali I.O. (2007). Structure, Thermal Behavior, Chemical Durability, and Optical Properties of the Na_2_O–Al_2_O_3_–TiO_2_–Nb_2_O_5_–P_2_O_5_ Glass System. J. Am. Ceram. Soc..

[48] Chrissanthopoulos A., Pouchan C., Papatheodorou G.N. (2001). Structural Investigation of Vanadium-Sodium Metaphosphate Glasses. Zeitschrift für Naturforschung A.

[49] Hejda P., Holubová J., Černošek Z., Černošková E. (2017). The Structure and Properties of Vanadium Zinc Phosphate Glasses. J. Non-Cryst. Solids.

[50] Du M., Huang K., Guo Y., Xie Z., Jiang H., Li C., Chen Y. (2019). High Specific Capacity Lithium Ion Battery Cathode Material Prepared by Synthesizing Vanadate–Phosphate Glass in Reducing Atmosphere. J. Power Sources.

[51] Zhao Z., Gao X., Wachs I.E. (2003). Comparative Study of Bulk and Supported V−Mo−Te−Nb−O Mixed Metal Oxide Catalysts for Oxidative Dehydrogenation of Propane to Propylene. J. Phys. Chem. B.

[52] Srikumar T., Srinvasa Rao C., Gandhi Y., Venkatramaiah N., Ravikumar V., Veeraiah N. (2011). Microstructural, Dielectric and Spectroscopic Properties of Li_2_O–Nb_2_O_5_–ZrO_2_–SiO_2_ Glass System Crystallized with V_2_O_5_. J. Phys. Chem. Solids.

[53] Rao K.J., Sobha K.C., Kumar S. (2001). Infrared and Raman Spectroscopic Studies of Glasses with NASICON-Type Chemistry. J. Chem. Sci..

[54] Ferreira B., Fargin E., Manaud J.P., Flem G.L., Rodriguez V., Buffeteau T. (2004). Second Harmonic Generation Induced by Poling in Borophosphate Bulk and Thin Film Glasses. J. Non-Cryst. Solids.

[55] Pereira R.R., Aquino F.T., Ferrier A., Goldner P., Gonçalves R.R. (2016). Nanostructured Rare Earth Doped Nb_2_O_5_: Structural, Optical Properties and Their Correlation with Photonic Applications. J. Lumin..

[56] Ardelean I., Rusu D., Andronache C., Ciobotă V. (2007). Raman Study of *x*MeO·(100−*x*)[P_2_O_5_·Li_2_O] (MeO ⇒ Fe_2_O_3_ or V_2_O_5_) Glass Systems. Mater. Lett..

[57] Dimitrov V., Dimitriev Y. (1990). Structure of Glasses in PbO-V_2_O_5_ System. J. Non-Cryst. Solids.

[58] Hayakawa S., Yoko T., Sakka S. (1995). IR and NMR Structural Studies on Lead Vanadate Glasses. J. Non-Cryst. Solids.

[59] Assem E.E., Elmehasseb I. (2011). Structure, Magnetic, and Electrical Studies on Vanadium Phosphate Glasses Containing Different Oxides. J. Mater. Sci..

[60] Rair D., Rochdi A., Majjane A., Jermoumi T., Chahine A., Touhami M.E. (2016). Synthesis and Study by FTIR, ^31^P NMR and Electrochemical Impedance Spectroscopy of Vanadium Zinc Phosphate Glasses Prepared by Sol–Gel Route. J. Non-Cryst. Solids.

[61] Komatsu T., Honma T., Tasheva T., Dimitrov V. (2022). Structural Role of Nb_2_O_5_ in Glass-Forming Ability, Electronic Polarizability and Nanocrystallization in Glasses: A Review. J. Non-Cryst. Solids.

[62] Moustafa Y.M., El-Egili K. (1998). Infrared Spectra of Sodium Phosphate Glasses. J. Non-Cryst. Solids.

[63] Muñoz F., Rocherullé J., Ahmed I., Hu L., Musgraves J.D., Hu J., Calvez L. (2019). Phosphate Glasses. Springer Handbook of Glass.

[64] Tricot G., Montagne L., Delevoye L., Palavit G., Kostoj V. (2004). Redox and Structure of Sodium-Vanadophosphate Glasses. J. Non-Cryst. Solids.

[65] Tricot G., Vezin H. (2013). Description of the Intermediate Length Scale Structural Motifs in Sodium Vanado-Phosphate Glasses by Magnetic Resonance Spectroscopies. J. Phys. Chem. C.

[66] Duffy J.A. (1996). Redox Equilibria in Glass. J. Non-Cryst. Solids.

[67] Murawski L., Chung C.H., Mackenzie J.D. (1979). Electrical Properties of Semiconducting Oxide Glasses. J. Non-Cryst. Solids.

[68] Saiko I.A., Saetova N.S., Raskovalov A.A., Il’ina E.A., Molchanova N.G., Kadyrova N.I. (2020). Hopping Conductivity in V_2_O_5_-P_2_O_5_ Glasses: Experiment and Non-Constant Force Field Molecular Dynamics. Solid State Ion..

[69] Razum M., Pavić L., Pajić D., Pisk J., Mošner P., Koudelka L., Šantić A. (2024). Casting a New Light on the Polaronic Transport in Vanadate-Phosphate Glasses. J. Am. Ceram. Soc..

[70] Summerfield S. (1985). Universal Low-Frequency Behaviour in the a.c. Hopping Conductivity of Disordered Systems. Philos. Mag. B.

[71] Meyer W.V., Neldel H. (1937). Über die Beziehungen Zwischen der Energiekonstanten und der Mengenkonstanten a in der Leitwerts Temperaturformel Bei Oxydischen Halbleitern. Z. Tech. Phys..

[72] De La Torre A.G., Bruque S., Aranda M.A.G. (2001). Rietveld Quantitative Amorphous Content Analysis. J. Appl. Cryst..

