# Supplementary material for: Exploring the Effect of V2O5 and Nb2O5 Content on the Structural, Thermal, and Electrical Characteristics of Sodium Phosphate Glasses and Glass–Ceramics
Source: Int J Mol Sci. 2024 Mar 5;25(5):3005. doi: 10.3390/ijms25053005 (PMC10932346; doi:10.3390/ijms25053005)
Supplement: Supplementary file 1 [file ijms-25-03005-s001.zip › ijms-2897779-supplementary.pdf]

International Journal of *Molecular Sciences*

MDPI

Supplementary Materials

Exploring the Effect of V_2O_5 and Nb_2O_5 Content on the Structural, Thermal, and Electrical Characteristics of Sodium Phosphate Glasses-(Ceramics)

Sara Marijan [1], Marija Mirosavljević [1], Teodoro Klaser [1], Petr Mošner [2], Ladislav Koudelka [2], Željko Skoko [3], Jana Pisk [4] and Luka Pavić [1,*]

1. SEM-EDS analysis

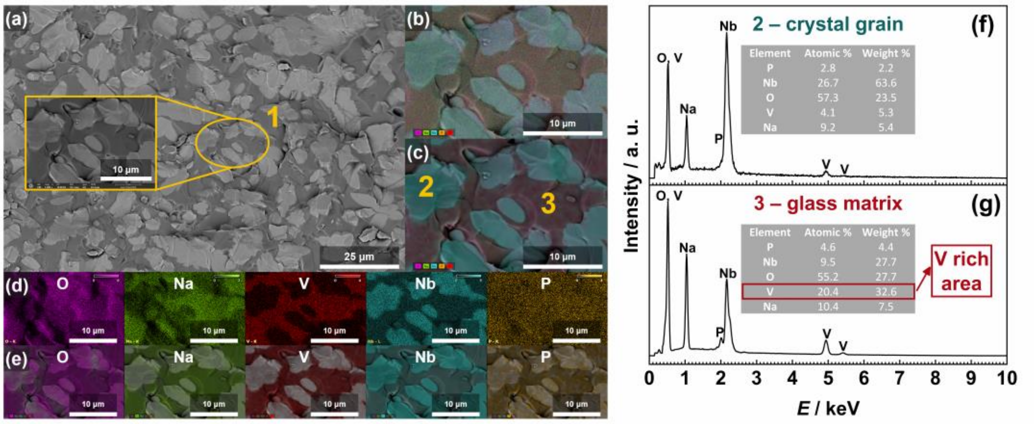

Figure S1. (a) SEM micrographs of 25V-35Nb glass-ceramic, (b) count X-ray mapping, (c) quantitative X-ray mapping, (d) count and (e) quantitative elemental mapping of O, Na, V, Nb and P from a selected area of the given glass-ceramic, (f, g) EDS spectra from selected areas of 25V-35Nb glass-ceramic.

2. Vibrational spectroscopy – *Raman* and IR-ATR studies

Table S1. Assignment of Raman and IR-ATR bands.

ν (cm^{-1})				Assignment
Raman	Ref.	IR-ATR	Ref.	
60–90	[1]	-	-	translational modes of the Na^+ cation
240–280	[2–4]	-	-	distortion of octahedral NbO_6
270–285, 365–380	[2–5]	400–600	[6,7]	δ(O=P–O) and δ(O–P–O)
450	[1,2,4,8]	420–530	[6,9,10]	P–O–Nb bonding
450–550	[24]	450–550	[18,25–28]	ν(V–O–V) in metavanadates
555, 605	[1,2,4,8]	530–665	[6,9,10]	coupled vibrational modes of intermediate Nb–O and O–P–O bonds
630–660	[2–5,8,11–14]	630–660	[13]	ν(Nb–O) in 3D network of slightly distorted octahedral NbO_6 units shared via corners
695	[2–5,8,11]	675–715	[6–8,15]	ν_s(P–O–P), Q^2
640	[16,17]	640	[18]	P–O–V bonding
735	[2–5,8,11]	715–755	[6,8,15]	ν_s(P–O–P), Q^1
600–700	[19]	600–700	[20]	ν(V–O–Nb)
740–770	[2–5,8,11–14]	760	[13]	ν(Nb–O–Nb) in corner-shared NbO_6 octahedral units
755–780	[16]	755–800	[18]	ν(V–O–V) bridging bond
-	-	880–920	[7,8,14,21,22]	ν_{as}(P–O–P)
850	[2–5,8,11–14]	830–860	[13,23]	ν(Nb–O–Nb) in NbO_6 octahedra linked into chains
945–965	[2–5,8,11–14]	925–975	[13,23]	ν(Nb–O) in highly distorted octahedral NbO_6 units
955–975	[24]	950–990	[18,25–28]	ν(V=O)
975–995	[4,8,11]	975–995	[6,8]	ν_s (PO_4^{3-}), Q^0
1045–1075	[4,8,11]	1045–1075	[6,8,14,22]	ν_{as} (PO_4^{3-}), Q^0
1085–1125	[2–4,8,11]	1085–1125	[8,21,22]	ν_s (PO_3^{2-}), Q^1
1135–1155	[2–4,8,11]	1135–1155	[8,22]	ν_s (PO_2^{-}), Q^2
1185–1225	[2–4,8,11]	1185–1225	[8,14,21,22]	ν_{as} (PO_3^{2-}), Q^1
1225–1275	[2–4,8,11]	1225–1275	[7,8,22]	ν_{as} (PO_2^{-}), Q^2

2. Scaling properties of conductivity spectra

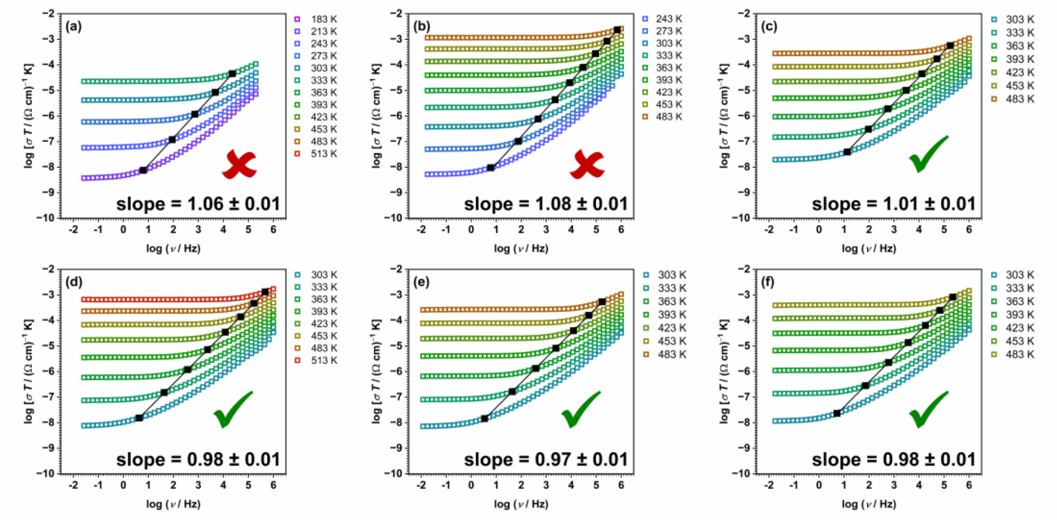

Figure S2. Conductivity spectra of (b) 25V-0Nb, (c) 25V-5Nb, (d) 25V-10Nb, (e) 25V-15Nb, (f) 25V-20Nb and (g) 25V-25Nb glass samples. The onset frequencies of conductivity dispersion, as defined by the equation $\sigma'(\nu_0) = 2\sigma_{DC}$, are represented by full black square symbols, while the straight black lines are obtained by linear regression of the $\log(\sigma' T)$ vs $\log\nu_0$ dependence.

References

1. Benyounoussy, S.; Bih, L.; Muñoz, F.; Rubio-Marcos, F.; Naji, M.; El Bouari, A. Structure, Dielectric, and Energy Storage Behaviors of the Lossy Glass-Ceramics Obtained from Na_2O-Nb_2O_5-P_2O_5 Glassy-System. *Phase Transit.* **2021**, *94*, 634–650. https://doi.org/10.1080/01411594.2021.1949458.
2. Flambard, A.; Videau, J.J.; Delevoye, L.; Cardinal, T.; Labrugère, C.; Rivero, C.A.; Couzi, M.; Montagne, L. Structure and Non-linear Optical Properties of Sodium–Niobium Phosphate Glasses. *J. Non-Cryst. Solids* **2008**, *354*, 3540–3547. https://doi.org/10.1016/j.jnoncrysol.2008.03.017.
3. Benyounoussy, S.; Bih, L.; Muñoz, F.; Rubio-Marcos, F.; EL Bouari, A. Effect of the Na_2O–Nb_2O_5–P_2O_5 Glass Additive on the Structure, Dielectric and Energy Storage Performances of Sodium Niobate Ceramics. *Heliyon* **2021**, *7*, e07113, https://doi.org/10.1016/j.heliyon.2021.e07113.
4. Razum, M.; Pavić, L.; Ghussn, L.; Moguš-Milanković, A.; Šantić, A. Transport of Potassium Ions in Niobium Phosphate Glasses. *J. Am. Ceram. Soc.* **2021**, *104*, 4669–4678, https://doi.org/10.1111/jace.17882.
5. Koudelka, L.; Kalenda, P.; Mošner, P.; Montagne, L.; Revel, B. Potassium Niobato-Phosphate Glasses and Glass-Ceramics. *J. Non-Cryst. Solids* **2021**, *572*, 121091. https://doi.org/10.1016/j.jnoncrysol.2021.121091.
6. Rambo, C.R.; Ghussn, L.; Sene, F.F.; Martinelli, J.R. Manufacturing of Porous Niobium Phosphate Glasses. *J. Non-Cryst. Solids* **2006**, *352*, 3739–3743, https://doi.org/10.1016/j.jnoncrysol.2006.03.104.
7. Attafi, Y.; Liu, S. Conductivity and Dielectric Properties of Na_2O-K_2O-Nb_2O_5-P_2O_5 Glasses with Varying Amounts of Nb_2O_5. *J. Non-Cryst. Solids* **2016**, *447*, 74–79, https://doi.org/10.1016/j.jnoncrysol.2016.05.038.
8. Senapati, A.; Barik, S.K.; Venkata Krishnan, R.; Chakraborty, S.; Jena, H. Studies on Synthesis, Structural and Thermal Properties of Sodium Niobium Phosphate Glasses for Nuclear Waste Immobilization Applications. *J. Therm. Anal. Calorim.* **2023**, *148*, 355–369. https://doi.org/10.1007/s10973-022-11760-3.
9. Wang, B.; Greenblatt, M.; Yan, J. Ionic Conductivities of Crystalline and Glassy $Na_4NbP_3O_{12}$ and Crystalline $Na_6Nb_2P_6O_{23}$. *Solid State Ionics* **1994**, *69*, 85–89, https://doi.org/10.1016/0167-2738(94)90454-5.
10. Chu, C.M.; Wu, J.J.; Yung, S.W.; Chin, T.S.; Zhang, T.; Wu, F.B. Optical and Structural Properties of Sr–Nb–Phosphate Glasses. *J. Non-Cryst. Solids* **2011**, *357*, 939–945, https://doi.org/10.1016/j.jnoncrysol.2010.12.009.

11. Mošner, P.; Hostinský, T.; Koudelka, L. Thermal, Structural and Crystallization Study of Na_2O–P_2O_5–Nb_2O_5 Glasses. *J. Solid State Chem.* **2022**, *316*, 123545. https://doi.org/10.1016/j.jssc.2022.123545.
12. Komatsu, T.; Honma, T.; Tasheva, T.; Dimitrov, V. Structural Role of Nb_2O_5 in Glass-Forming Ability, Electronic Polarizability and Nanocrystallization in Glasses: A Review. *J. Non-Cryst. Solids* **2022**, *581*, 121414, https://doi.org/10.1016/j.jnoncrysol.2022.121414.
13. Iordanova, R.; Milanova, M.; Aleksandrov, L.; Shinozaki, K.; Komatsu, T. Structural Study of WO_3-La_2O_3-B_2O_3-Nb_2O_5 Glasses. *J. Non-Cryst. Solids* **2020**, *543*, 120132, https://doi.org/10.1016/j.jnoncrysol.2020.120132.
14. Karam, L.; Adamietz, F.; Rodriguez, V.; Bondu, F.; Lepicard, A.; Cardinal, T.; Fargin, E.; Richardson, K.; Dussauze, M. The Effect of the Sodium Content on the Structure and the Optical Properties of Thermally Poled Sodium and Niobium Borophosphate Glasses. *J. Appl. Phys. (Melville, NY, U. S.)* **2020**, *128*, 043106, https://doi.org/10.1063/5.0013383.
15. Teixeira, Z.; Alves, O.L.; Mazali, I.O. Structure, Thermal Behavior, Chemical Durability, and Optical Properties of the Na_2O–Al_2O_3–TiO_2–Nb_2O_5–P_2O_5 Glass System. *J. Am. Ceram. Soc.* **2007**, *90*, 256–263, https://doi.org/10.1111/j.1551-2916.2006.01399.x.
16. Chrissanthopoulos, A.; Pouchan, C.; Papatheodorou, G.N. Structural Investigation of Vanadium-Sodium Metaphosphate Glasses. *Zeitschrift für Naturforschung A* **2001**, *56*, 773–776, https://doi.org/10.1515/zna-2001-1114.
17. Hejda, P.; Holubová, J.; Černošek, Z.; Černošková, E. The Structure and Properties of Vanadium Zinc Phosphate Glasses. *J. Non-Cryst. Solids* **2017**, *462*, 65–71, https://doi.org/10.1016/j.jnoncrysol.2017.02.012.
18. Du, M.; Huang, K.; Guo, Y.; Xie, Z.; Jiang, H.; Li, C.; Chen, Y. High Specific Capacity Lithium Ion Battery Cathode Material Prepared by Synthesizing Vanadate–Phosphate Glass in Reducing Atmosphere. *J. Power Sources* **2019**, *424*, 91–99, https://doi.org/10.1016/j.jpowsour.2019.03.106.
19. Zhao, Z.; Gao, X.; Wachs, I.E. Comparative Study of Bulk and Supported V−Mo−Te−Nb−O Mixed Metal Oxide Catalysts for Oxidative Dehydrogenation of Propane to Propylene. *J. Phys. Chem. B* **2003**, *107*, 6333–6342, https://doi.org/10.1021/jp021640m.
20. Srikumar, T.; Srinvasa Rao, Ch.; Gandhi, Y.; Venkatramaiah, N.; Ravikumar, V.; Veeraiah, N. Microstructural, Dielectric and Spectroscopic Properties of Li_2O–Nb_2O_5–ZrO_2–SiO_2 Glass System Crystallized with V_2O_5. *J. Phys. Chem. Solids* **2011**, *72*, 190–200, https://doi.org/10.1016/j.jpcs.2010.12.009.
21. Rao, K.J.; Sobha, K.C.; Kumar, S. Infrared and Raman Spectroscopic Studies of Glasses with NASICON-Type Chemistry. *J. Chem. Sci. (Berlin, Ger.)* **2001**, *113*, 497–514, https://doi.org/10.1007/BF02708786.
22. Ferreira, B.; Fargin, E.; Manaud, J.P.; Flem, G.L.; Rodriguez, V.; Buffeteau, T. Second Harmonic Generation Induced by Poling in Borophosphate Bulk and Thin Film Glasses. *J. Non-Cryst. Solids* **2004**, *343*, 121–130, https://doi.org/10.1016/j.jnoncrysol.2004.07.010.
23. Pereira, R.R.; Aquino, F.T.; Ferrier, A.; Goldner, P.; Gonçalves, R.R. Nanostructured Rare Earth Doped Nb_2O_5: Structural, Optical Properties and Their Correlation with Photonic Applications. *J. Lumin.* **2016**, *170*, 707–717, https://doi.org/10.1016/j.jlumin.2015.08.068.
24. Ardelean, I.; Rusu, D.; Andronache, C.; Ciobotă, V. Raman Study of xMeO·(100−x)[P_2O_5·Li_2O] (MeO ⇒ Fe_2O_3 or V_2O_5) Glass Systems. *Mater. Lett.* **2007**, *61*, 3301–3304, https://doi.org/10.1016/j.matlet.2006.11.057.
25. Dimitrov, V.; Dimitriev, Y. Structure of Glasses in PbO-V_2O_5 System. *J. Non-Cryst. Solids* **1990**, *122*, 133–138, https://doi.org/10.1016/0022-3093(90)91058-Y.
26. Hayakawa, S.; Yoko, T.; Sakka, S. IR and NMR Structural Studies on Lead Vanadate Glasses. *J. Non-Cryst. Solids* **1995**, *183*, 73–84, https://doi.org/10.1016/0022-3093(94)00652-0.
27. Assem, E.E.; Elmehasseb, I. Structure, Magnetic, and Electrical Studies on Vanadium Phosphate Glasses Containing Different Oxides. *J. Mater. Sci.* **2011**, *46*, 2071–2076, https://doi.org/10.1007/s10853-010-5040-0.

28. Rair, D.; Rochdi, A.; Majjane, A.; Jermoumi, T.; Chahine, A.; Touhami, M.E. Synthesis and Study by FTIR, ^{31}P NMR and Electrochemical Impedance Spectroscopy of Vanadium Zinc Phosphate Glasses Prepared by Sol–Gel Route. *J. Non-Cryst. Solids* **2016**, *432*, 459–465, https://doi.org/10.1016/j.jnoncrysol.2015.11.001.

Disclaimer/Publisher's Note: The statements, opinions and data contained in all publications are solely those of the individual author(s) and contributor(s) and not of MDPI and/or the editor(s). MDPI and/or the editor(s) disclaim responsibility for any injury to people or property resulting from any ideas, methods, instructions or products referred to in the content.
